# Extensive Vibrational Characterisation and Long-Term Monitoring of Honeybee Dorso-Ventral Abdominal Vibration signals

**DOI:** 10.1038/s41598-018-32931-z

**Published:** 2018-10-01

**Authors:** M. Ramsey, M. Bencsik, M. I. Newton

**Affiliations:** Nottingham Trent University, School of Science and Technology, Clifton Lane, Clifton, Nottingham, NG11 8NS UK

## Abstract

A very common honeybee signal is the dorso-ventral abdominal vibration (DVAV) signal, widely accepted as a modulatory signal meaning: “prepare for greater activity”. In this study, using ultra-sensitive accelerometer technology embedded in the honeycomb, we visually confirm the one-to-one relationship between a DVAV signal being produced and the resulting accelerometer waveform, allowing the measurement of DVAV signals without relying on any visual inspection. We then demonstrate a novel method for the continuous *in-situ* non-invasive automated monitoring of this honeybee signal, not previously known to induce any vibration into the honeycomb, and most often inaudible to human hearing. We monitored a total of three hives in the UK and France, showing that the signal is very common, highly repeatable and occurs more frequently at night, exhibiting a distinct decrease in instances and increase in amplitude towards mid-afternoon. We also show an unprecedented increase in the cumulative amplitude of DVAV signals occurring in the hours preceding and following a primary swarm. We conclude that DVAV signals may have additional functions beyond solely being a foraging activation signal, and that the amplitude of the signal might be indicative of the switching of its purpose.

## Introduction

Eusociality is considered the most complex form of social structure, and is defined as the cooperative care of offspring born from reproductive individuals but reared by non-reproductive individuals with overlapping generations within a colony^1,2^. A fascinating model for this is the honeybee (*Apis mellifera*), for which ecological success relies heavily on division of labour into specialised behavioural groups (known as castes)^3^ that interact, forming a superorganism^4,5^. For example, upon returning to the hive, foragers deliver the nectar collected in the field to nectar receivers for processing and storage^6,7^. Nectar receiving is one task of the middle-aged caste whilst nectar retrieval is one of the forager caste, made up of the most mature individuals^8–12^. Honeybees will often switch between the tasks within their “task repertoire”^8,11,12^ depending on specific conditions within the hive (e.g. a sudden influx of nectar). Coordination of these are mediated through many cues and signals produced by individuals of the colony^7,10,13^. One fundamental signal investigated by numerous past researchers is the honeybee Dorso-Ventral Abdominal vibration signal (or DVAV signal).

The DVAV signal was first described by Haydak^14^ and since has been referred to under many different names: “jerking dance”^15^, “vibration signal”, “vibration dance”^16^, “shaking signal”^17^, “DVA-V signal”^18^. For the purpose of this paper, however, the signal will retain the name honeybee dorso-ventral abdominal vibration signal (or DVAV signal) as it unambiguously points to the signal that we are focussing on. For the full list of alternative names, see Schneider and Lewis^19^.

As with the choice of name, the function of the DVAV signal also causes debate amongst scientists. However, it is generally recognised as having a modulatory function^20^. Hölldobler and Wilson^21^ define modulatory signals as *inefficient communication pathways that influence the behaviour of receivers*, *not through direct instruction but by slightly shifting the probability of the performance of other behaviours*. Distinctive behavioural responses are hard to establish in modulatory signals making them difficult to associate with any explicit function^22^. Owing to their non-specificity, modulatory signals can act upon many different individuals; altering the performance of numerous different contemporaneous activities^19^.

When a honeybee performs a DVAV signal on another bee, it does this by gripping the comb with its metathoracic legs, the receiver with its prothoracic and mesothoracic legs, and then vigorously and rhythmically shaking its abdomen in a direction normal to the plane of the honeycomb^22,23^ for 0.9 to 1.5 seconds, producing vibrations at 10 to 22 Hz^24^. During the shaking, the signal-receiving bee remains in one spot upon the comb, appears amenable, and only moves its body in response to that of the shaker^22^. A video of the DVAV signal is provided in Video S1. It has been demonstrated in numerous studies that (i) one given honeybee will produce this signal on multiple individuals concurrently, and (ii) a bee will also often deliver DVAV signals directly onto the honeycomb too. In addition, honeybees producing DVAV signals often do so as part of “shaking runs” (Video S2), in which they roam over large areas of the hive, producing a series of these signals (up to 20 or more per min) that can last from several minutes to over an hour^16,19,22,23^.

Even though only around 13% of workers ever perform DVAV signals during their lifetimes^25,26^, it has been suggested that the DVAV signal can be observed hundreds of times per hour, usually regulating two distinct colony activities: foraging and swarming^27^. The signallers tend to be the older workers within the foraging caste^24–26^. However, it has been shown that even two day old worker bees can perform DVAV signals on fellow workers, as well as on queens and on queen cells^24,26–28^. Duong and Schneider^29^ suggested that an individual’s genetics may also influence their potential to perform a DVAV signal, using colonies of isolated patrilines where vast differences in DVAV signal production was observed. They also showed that the production of DVAV signals usually begins three to five days before waggle dancing with some individuals performing it persistently for up to nine days. Upon receiving a DVAV signal, it has been proposed that the recipient will increase their speed of movement within the hive, particularly towards the location of waggle dancers, and increase the overall rate of any hive-based activity they are engaged in^16,30^. After a few days of successful foraging, it has also been suggested that there is an increase in the number of DVAVs acting within a colony in the early morning before foraging begins and in the late afternoon after foraging has ceased^22^. It has been shown that there are seasonal peaks in DVAV signal production associated with peak foraging times. Seeley *et al*.^23^ showed that individuals returning from their first few trips to a newly found resource patch would only produce DVAV signals within the hive. Further successive trips to the new forage patch resulted in signallers gradually transitioning from DVAV signalling to waggle dancing until only waggle dances were eventually seen.

Outside the remit of foraging and swarming, the signal can be received by, and influence the behaviour of all ages of workers. For example, Schneider^20^ and Hyland *et al*.^30^ show that recipients of the signal that are of nursing age (young workers) show significant increases in brood care. The signal causes a non-specific increase in activity in all workers expressed as increased locomotion, cell inspection and trophallactic activity, which facilitates the acquisition of information. This, in turn, simultaneously enhances the delivery of many different tasks depending upon recipient age and physiological state, including brood care, food processing, and foraging^16,20,30–32^.

The individuals of the worker population are not the only intended recipients of DVAV signals. Queens have also been observed receiving DVAV signals from worker bees. Allen^17,33,34^ and Hammann^35^ noted that honeybees tended to DVAV signal their queens before she was about to leave the hive, taking flight either with a swarm or to mate. With regards to virgin queens, the rate of signalling that they receive has been shown to be positively correlated with fighting success and survival^36–38^. Fletcher^39–41^ also suggested that the rate of which a mated queen receives DVAV signals rises rapidly once queen rearing begins and drops off a few hours prior to swarming. Similarly, Peirce *et al*.^42^ also observed that DVAV signal production on queens and fellow workers increases in the two to three days preceding swarming. This evidence all supports the message of “prepare for flight^43^ or greater activity^23^”. Additionally, drones also have been observed to be DVAV signals recipients^44^. It is believed that DVAV signalling plays a role in drone maturation and maintenance by making them more active within the hive and thus more likely to receive the care (grooming and trophallaxis) required for sexual development.

The vast majority of studies on this signal, however, focus on its occurrences within worker-worker interactions, as these are the most common recipients. Early studies suggested it had a function within the foraging domain^24,29^ but convincing evidence was lacking. The extensive work of Schneider and various colleagues^16,19,43,45^, later supported by Neih^22^ and Seeley *et al*.^23^, provided evidence that it is a signal that conveyed the message “prepare for increased activity levels”. This means that the colony is ready to make best use of an imminent energetically expensive opportunity, such as a high-level forage influx.

The wealth of previous studies shows that the specific function of the DVAV signal is difficult to pinpoint using manipulative behavioural experiments. By nature, behavioural studies are biased towards a specific experimental hypothesis; scientists come up with a theory and undertake analysis of specific manipulations to test whether or not it is accurate, and in the majority of cases, this is highly suitable. However, this sometimes leads to misinterpretations upon examination of the bigger picture^46^. For example, the same honeybee signal can have different connotations depending on the conditions associated with its production; assigning one definition is therefore inappropriate in most circumstances. Exploration studies based on continuous long-term statistics recorded *in situ* also have their drawbacks in that you cannot observe the exact visual behavioural information of the recipient or the receiver during the production of individual signals, for example. However, they can contribute a wealth of information, not available through behavioural studies (such as, continuous recordings from colonies experiencing natural conditions), that can further the understanding of honeybee communication. The DVAV is well known by visual inspections but monitoring with durable in-hive vibrational sensors would allow continuous measurements that do not require invasive visual inspections. The aim of this study was therefore to develop a method to physically characterise and find unique DVAV signal features for continuously monitoring the honeybee DVAV signal non-invasively on the long-term.

Unlike previous work into the long-term monitoring of honeybee signals^47,48^, where potentially unintentional signals have been monitored within a honeybee hive, we are here providing a breakthrough in the long-term monitoring of a signal that is highly meaningful to honeybees, which may provide an indirect assessment of the status of the honeycomb, specific information on health disorders and estimates on a colony’s intention to swarm.

The outcomes of this extensive monitoring will allow us to discuss results (1) in terms of any evidence of long-term trends; (2) in the context of the colony status; (3) measured from multiple colonies in the UK and France, one of which includes a primary swarm and two secondary swarms, one that succumbed to disease and one that was measured at the periphery of a colony that superseded its queen. In light of previous authors work, our analysis of daily and long-term statistics should reveal that (1) more signals are produced in the morning with a decrease towards the afternoon; (2) more signals are produced in the spring and summer as opposed to the winter; and (3) more signals will be recorded at times when more foragers will be in the hive, at night and during times of heavy rain, for example.

## Results

### Vibrational quantitation of DVAV signals

**Figure 1:**
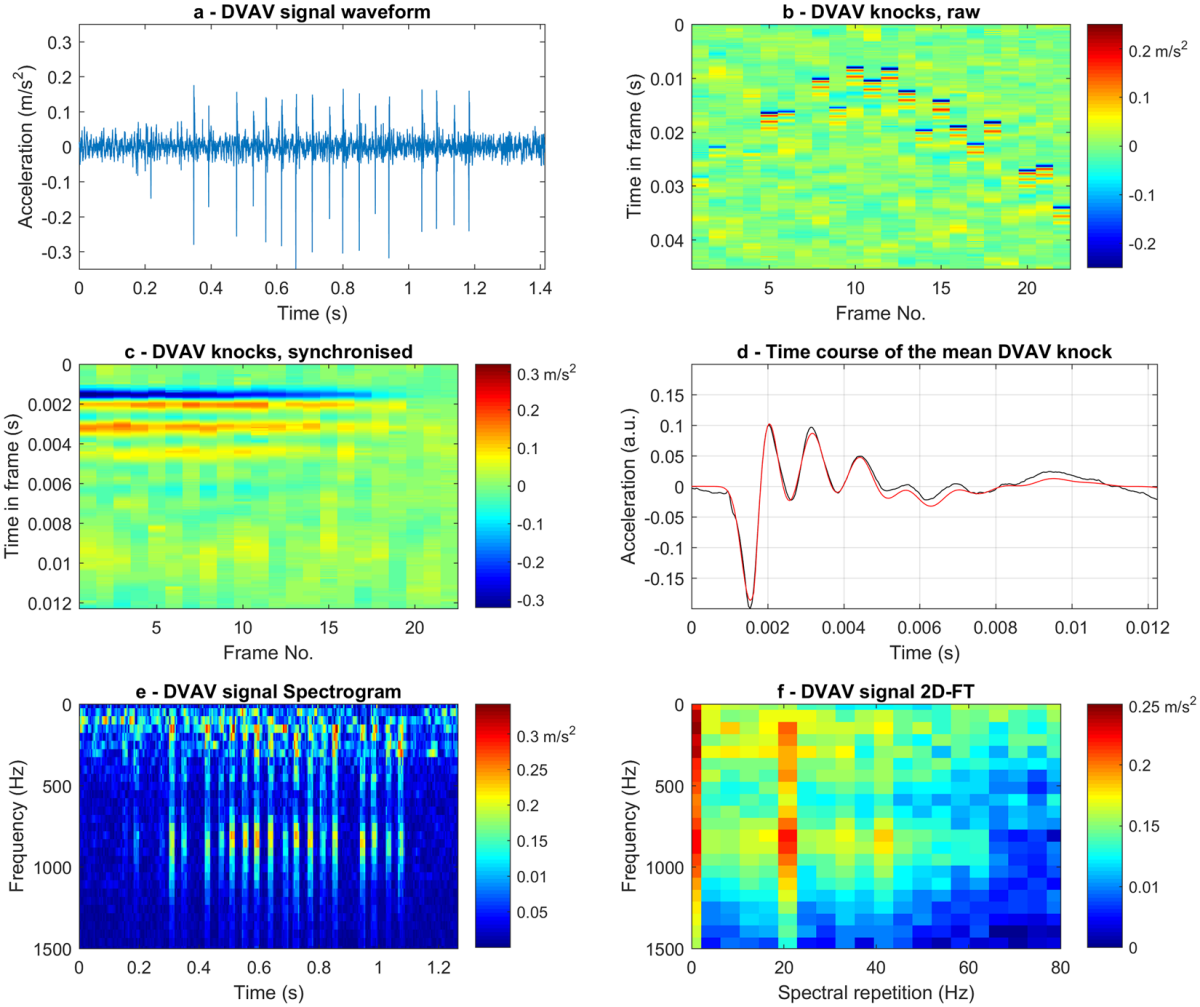
Vibrational properties of a DVAV signal delivered onto an accelerometer. (**a**) Time course of honeycomb acceleration waveform during the delivery of a DVAV signal; (**b**) The same waveform, shown in successive frames adjusted to the time gap residing between two individual abdominal-honeycomb collisions; (**c**) The DVAV signal knocks, aligned to the first one; (**d**) The time course (black) of the mean of the DVAV signal knocks, and fitted curve (red); (**e**) The spectrogram of the complex DVAV waveform in Fig. 1a; (**f**) The 2D-FT image of the complex DVAV waveform in Fig. 1a. The colour bar displays the linear scale amplitude in m/s^2^. The colour bar displays the linear scale amplitude in m/s^2^.

The physical properties of a specific DVAV signal that was delivered directly onto one of our accelerometers (Video S3), parallel to the axis of the sensor in a positive direction, can be found in Fig. 1. For comparison between a DVAV signal that occurred on the same side of the frame as the one shown in Fig. 1, which is seen in Video S3, and on the other side of the frame that is seen in Video S4, refer to Supplementary Figs S1 and S2. This particular signal has a duration of 1.1 s, a fundamental frequency of 22.1 Hz (Fig. 1a) and a mean peak acceleration of 0.162 m/s^2^ (Fig. 1d). Figure 1b demonstrates the same DVAV signal recording, which has been segregated into equal length windows called frames around each of the individual “knocks”, revealing the characteristic “П-shape”, shown in synchrony with the pulse in video S3, which is unique and common to all of these signals (see subplot b throughout Video S5). This phenomenon highlights the initial increase and later decrease in the rate at which the honeybee delivers its abdominal knocks during signal production, with the longest part of the signal, in between, exhibiting a constant rate of delivery. These knocks are then aligned to each other in Fig. 1c and averaged in Fig. 1d to show how, following one abdominal knock, the honeycomb reacts and relaxes with typical time decays that are orders of magnitude longer than the duration of the actual bee to comb collision. There is a sharp burst of negative acceleration consistent with the downward movement of the honeybee’s abdomen, which is followed by oscillations that gradually decrease in amplitude as the honeycomb returns to a state of equilibrium. It is also apparent that the honeycomb fully reaches it equilibrium before the next abdominal knock commences. The bee abdominal collision in Fig. 1d (black curve) is well described (red curve) by a Gaussian peak with amplitude 0.2 m/s^2^, full width at half-maximum 0.56 ms, and centred at 1.54 ms. The oscillations taking place in the honeycomb (black curve) immediately after the knock are well described (red curve) by the sum of three exponentially decaying sinusoidal functions. The parameters of these oscillations will change with the local honeycomb load. For this particular signal, the first component is a fast oscillation with an amplitude of 0.2 m/s^2^, a frequency of 808 Hz and a decay constant of 2 ms. The next is a mid-range oscillation with an amplitude of 0.0253 m/s^2^, a frequency of 135 Hz and a 2.8 μs decay constant. Finally there is the third and slowest oscillation with an amplitude of 0.0266 m/s^2^, a frequency of 60 Hz and a 2 ms decay constant. This relaxation curve gives a remarkable insight into the local natural vibrational modes of the honeycomb. Due to the honeybee only being able to hit the honeycomb (it cannot pull on it effectively), for DVAV signals that occur along the axis of the accelerometer, the original sharp knocks are comprised of negative signals whilst the honeycomb relaxation wave is balanced around zero. However, as demonstrated in Supplementary Fig. S2, if the DVAV signal is delivered on the other side of the frame, as in video S4, the sharp bursts will comprise of a positive signal. This unbalanced feature is typical of honeybee signals originally delivered as vibrations (as opposed to sounds) and is also present in the waveform of most whooping signals^48^.

In many instances, including this one, the honeycomb relaxation can be observed even on the individual knocks (Fig. 1c), although it is much clearer on the averaged knock (Fig. 1d). To see a large collection of analysed DVAV signals with their corresponding audible accelerometer data, see Video S5. Included in Supplementary Fig. S3 is this analysis for a commonly occurring honeybee signal from within the hive, which we call the “high-amplitude clicks” where spikes similar to those seen in DVAV signals are observed. In Supplementary Fig. S3b, we see in the case of high-amplitude clicks present throughout our datasets that the spikes in the waveform occur much less frequently and more sporadically than in the case of the DVAV signal and do not have the characteristic П-shape over a one second excerpt. These differing features are exploited in our data mining strategy in order to discriminate them effectively.

Statistics were undertaken on the accelerometer trace of 27 high-SNR DVAV signals that all underwent the same physical characterisation as the pulse featured in Fig. 1. All descriptive statistics are given in Table 1 for the frequency, the duration, the number of abdominal knocks and the time difference between two consecutive knocks to give the reader insight into the distribution of data for each. In Table 1, it is shown that the physical characteristics of the twenty-seven high-SNR DVAV signals are consistent with the of those of the signals that were captured by video analysis (Videos S3 and S4) as well as those published by Gahl^24^, highlighting the repeatability of this signal and further strengthening the fact that we have a one-to-one relationship between a DVAV signal and its accelerometer trace. Full characterisation of these twenty-seven signals along with corresponding audio has been made available in Video S5.

**Table 1:** The statistics of the vibration properties of 27 audible DVAV signals extracted by our software.

	Mean	Mode	Median	Min	Max	Skew	Kurtosis
Frequency (Hz)	18.21	16.11	17.89	13.1	22.75	0.065	2.67
Duration (s)	1.01	0.92	0.99	0.49	1.68	0.46	2.54
Number of knocks	17.92	17.92	18	8	27	−0.12	2.27
Time between two knocks (s)	0.0056	0.0054	0.05	0.044	0.065	0.84	4.2

Spectral analysis was then undertaken on the signal to see what further information could be revealed pertaining to the periodic nature of the time course of the DVAV signal complex waveform. From Fig. 1e, it can be seen that that the high-amplitude DVAV signal displayed in Fig. 1a consists of 22 regular knocks that occur over one second with an average peak acceleration of 0.162 m/s^2^. On the spectrogram these are seen between 0.2 and 1.2 seconds as a series of sharp regular broadband spectra but without quantitative information about their periodicity, which is the feature that is most specific to the signal we are interested in. The 2-dimensional Fourier Transform (2D-FT) further computes the frequency of repeating spectra over a set time period (see Methods), and is a very powerful method that is remarkably well suited for the analysis of DVAV signals because each knock regularly produces a highly repeatable broadband of frequencies, due to the very sharp nature of any knock (Fig. 1f, Supplementary Fig. S4). The 2D-FT is calculated and displayed in synchrony for this DVAV signal in Video S3 and for another one occurring on the other side of the frame in Video S4. In Fig. 1f, 2D-FT analysis of this signal shows a broadband spectrum between 0 and 1300 Hz that is repeated 22 times a second. This spectral repetition then has two harmonics, one harmonic at twice and another low amplitude harmonic at three times the fundamental frequency. The signal at higher harmonics are partly due to the fact that the second FFT is done on a power spectrum, rather than on a complex data set, and partly due to the temporal sharpness of the spectra on the individual knocks. The information found within the 0 Hz spectral repetition frequency originates from the fact that all data is positive in the spectrogram. To showcase the significance of the 2D-FT as a tool for the analysis of DVAV signals, in subplot e of the Video S5 the 2D-FT of the 27 high-SNR DVAV signals are displayed with audio.

In Supplementary Fig. S4, the power spectra of each averaged knock of the twenty-seven high SNR pulses are shown. Principal component analysis was used to order the pulses by decreasing distance to the population mean. It can be seen that the majority of pulses have a sharp peak at around 60 Hz, characteristic of a slow oscillation, and a secondary broadband peak, that is characteristic of an ultra-fast oscillation, between 500 and 1000 Hz. In line with the above, there appears to be little relevant information above 1500 Hz for the majority of DVAV signals. The polarity of the pulses, caused by the side of the frame upon which the signal was delivered, appears to have no effect on the spectra of the pulse’s mean knock. Figure S4 also shows that the four DVAV signals that come from other hives to that of the majority are outliers to the rest of the population.

**Figure 2:**
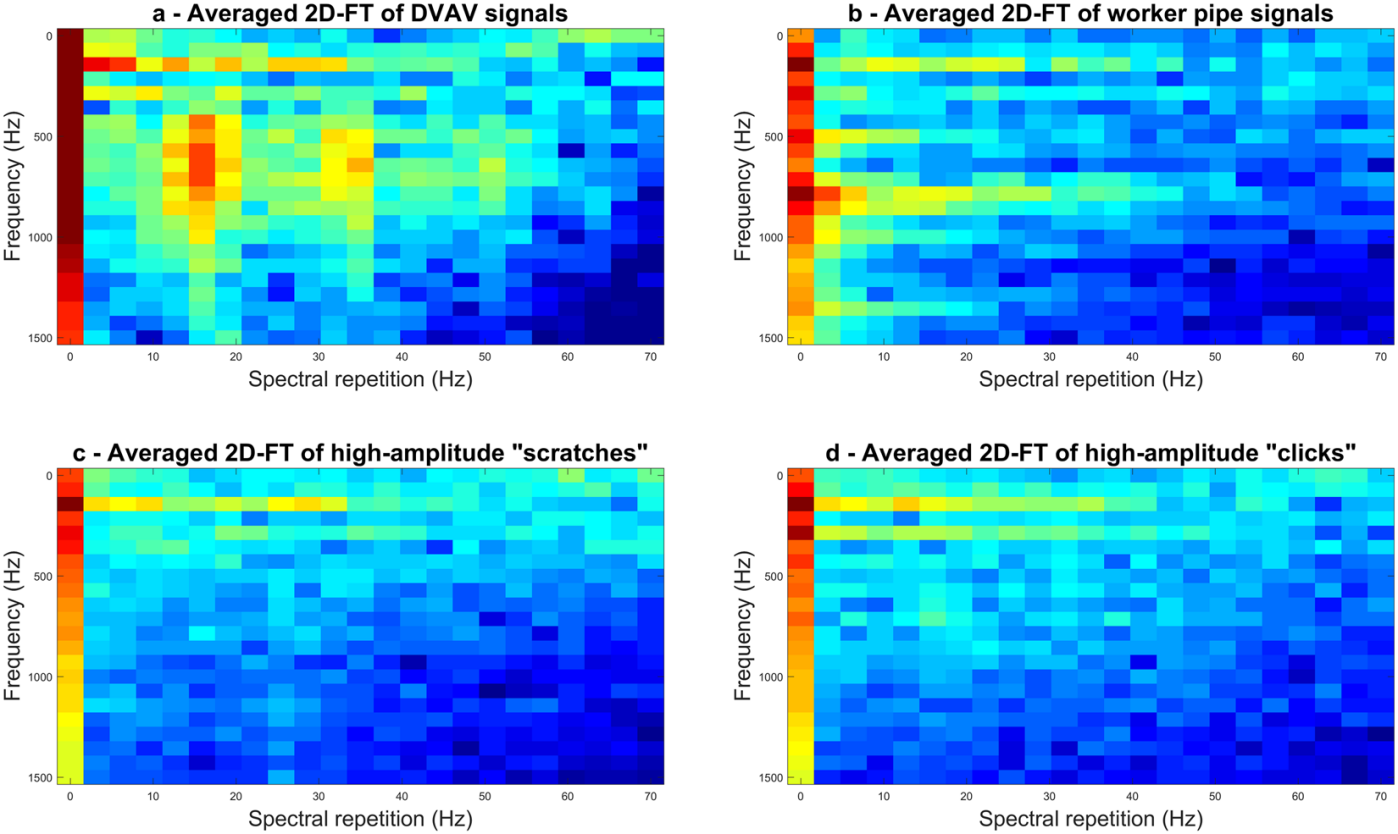
The averaged 2D-FT calculated on 150 examples of the signal waveform of commonly encountered pulsed vibrations (**a**) DVAV signals, (**b**) worker pipes, (**c**) wax scratching, and (**d**) high-amplitude “click” vibrations.

To demonstrate the strength of the method relying on the use of the 2D-FT, Fig. 2 shows a comparison of the 2D-FT with other one-second-long signals that can be found within our datasets. It is seen in Fig. 2a that the averaged 2D-FT of 150 examples of extracted DVAV signal waveforms exhibits broadband vertical peaks at around 18 Hz, 32 Hz and 45 Hz (second and third harmonics). For comparison, in Fig. 2b–d, an averaged 2D-FT of worker pipes, high-amplitude scratching sounds and high-amplitude clicks averaged over 150 examples for each are also displayed. It is apparent from these that well-defined vertical broadbands are unique to DVAV signals at these frequencies, and this information is utilised within the first pass of our detection software. The 2DFT works so well for the DVAV signal over the other signals owing to the regularity of the sharp individual knocks over a one-second time course. It is seen that all 2DFT images exhibit horizontal broadband peaks at 125 Hz and 250 Hz that can be attributed to the background sound of the buzzing of the wings (e.g. see Ramsey *et al*.^48^). The worker pipes (Fig. 2b) exhibit horizontal peaks at 500 Hz and 800 Hz. No definitive information can be seen for the high-amplitude “scratching” in Fig. 2c or the high-amplitude “clicks” presented in Fig. 2d, probably due to averaged images that do not exhibit repeatable features.

### The spatial distribution of DVAV signals

A total of 26 videos representing a total time duration of 8 hours of recording were visually examined to detect the actual number and location of DVAV signals that occurred on both sides of the focal frame (see Methods). As all assessments showed a similar spatial distribution, four specific examples are presented below to give a representation of the distribution over each season.

**Figure 3:**
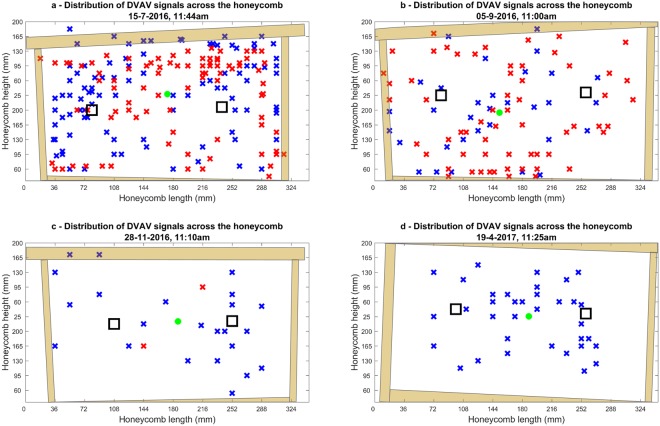
The spatial analysis of DVAV signals occurring across the honeycomb recorded over a series of 10-minutes of videos recorded in (**a**) July 2016, (**b**) September 2016, (**c**) November 2016, and (**d**) April 2017. The large black squares indicate the location of the accelerometers. The blue and red X’s respectively indicate the location of DVAVs on the side of the honeycomb with visibly implanted accelerometers and on the other side, behind the accelerometers. Green dots signify the mean coordinates. The light brown shapes mark the location of the wooden frame surrounding the honeycomb as seen in the video footage.

In all 10-minute long videos sampled at various points throughout the year, we see from Fig. 3 an even distribution of DVAV signals across the honeycomb, regardless of the their total count captured on the film or the side they were recorded on. A full breakdown of the statistics corresponding to the plots in Fig. 3 is provided in Table 2 below.

**Table 2:** Tabulation of the spatial distribution results presented in Fig. 3.

Label	Fig. 3a	Fig. 3b	Fig. 3c	Fig. 3d
Date	15-07-2016	05-09-2016	28-11-2016	16-4-2017
Total DVAV signals	204	95	25	37
Side A	107	61	23	37
Side B	97	34	2	0

### Extensive visual inspection

Observations within this analysis suggests that the signaller makes full contact with the honeycomb in 33% of cases when performing a DVAV signal, regardless of its intended recipient (comb or conspecific). In the remaining instances (67%) where the abdomen did not make contact with the honeycomb, the DVAV signal was never detected. In Table 3 it can be seen that upon testing of our detection software with accelerometer data synchronous with video footage, the percentage of the overall detections that were genuine was confirmed by visual analysis to be 89%. Additionally, alteration to detection thresholds (see Methods) by making them more or less strict resulted in the increase in the number of false detections or dismissal of genuine DVAV signals, demonstrating that our original threshold is optimum. It can be seen from Table 3 that the overall number of accelerometer detections relative to the number of actual DVAV signals is around 2%. However, the percentage of detections overall appears to increase to 27% when the honeycomb frame-load is less dense (19^th^ April 2017, see Table 1 in Supplementary File S2). It can also be seen that videos captured around 2 pm produced the fewest DVAV signals and that DVAV signals can be observed in the winter when temperatures fall below 8 °C (see Table 1 in Supplementary File S2).

**Table 3:** The extensive visual analysis of DVAV signals occurring throughout videos recorded on both sides of the focal frame pertaining to the observation hive.

Date/Time of Video	Dur. (min)	Number of DVAVs Seen in Video	Number of Detected DVAVs using Software	Correct Detection Number	% of Genuine Detections	Abdomen Collisions: Bee/Comb
11:44 am 8^th^ July 2016	10	204	13	10	77	149/46
14:00 pm 21^st^ July 2016	10	10	0	0	100	10/0
12:05 pm 29^th^ July 2016	20	114	3	3	100	76/38
13:58 pm 19^th^ Aug 2016	20	19	1	1	100	15/4
14:31 pm 22^nd^ Aug 2016	20	0	0	0	100	0/0
16:00 pm 30^th^ Aug 2016	20	0	1	0	—	0/0
11:20 am 5^th^ Sep 2016	20	95	1	1	100	67/28
11:00 am 7th Sep 2016	20	30	0	0	100	22/8
13:01 pm 15^th^ Sep 2016	20	20	0	0	100	18/2
10:00 am 16^th^ Sep 2016	20	11	0	0	100	11/0
15:10 pm 28^th^ Nov 2016	20	25	0	0	100	13/8
16:21 pm 19^th^ April 2017	20	37	11	10	91	22/15
14:00 pm 9^th^ Aug 2017	10	0	0	0	100	0/0

The table features the number of actual DVAV signals that occurred, how many were identified by our detection software and the instances where the bee’s abdomen could be seen making contact with the honeycomb. For an extension of Table 2 that contains a full break down and detailed description of the videos analysed as part of this study, see Supplementary File S2.

### Long-term DVAV signal statistics

The results displayed in this section are those obtained from a hive known as the ‘2015 French hive’ and is supported by the results of two more datasets: the ‘Clifton Observation hive’ and the ‘2017 French hive’, which can be found in the supplementary material. For information pertaining to these hives, see Methods.

**Figure 4:**
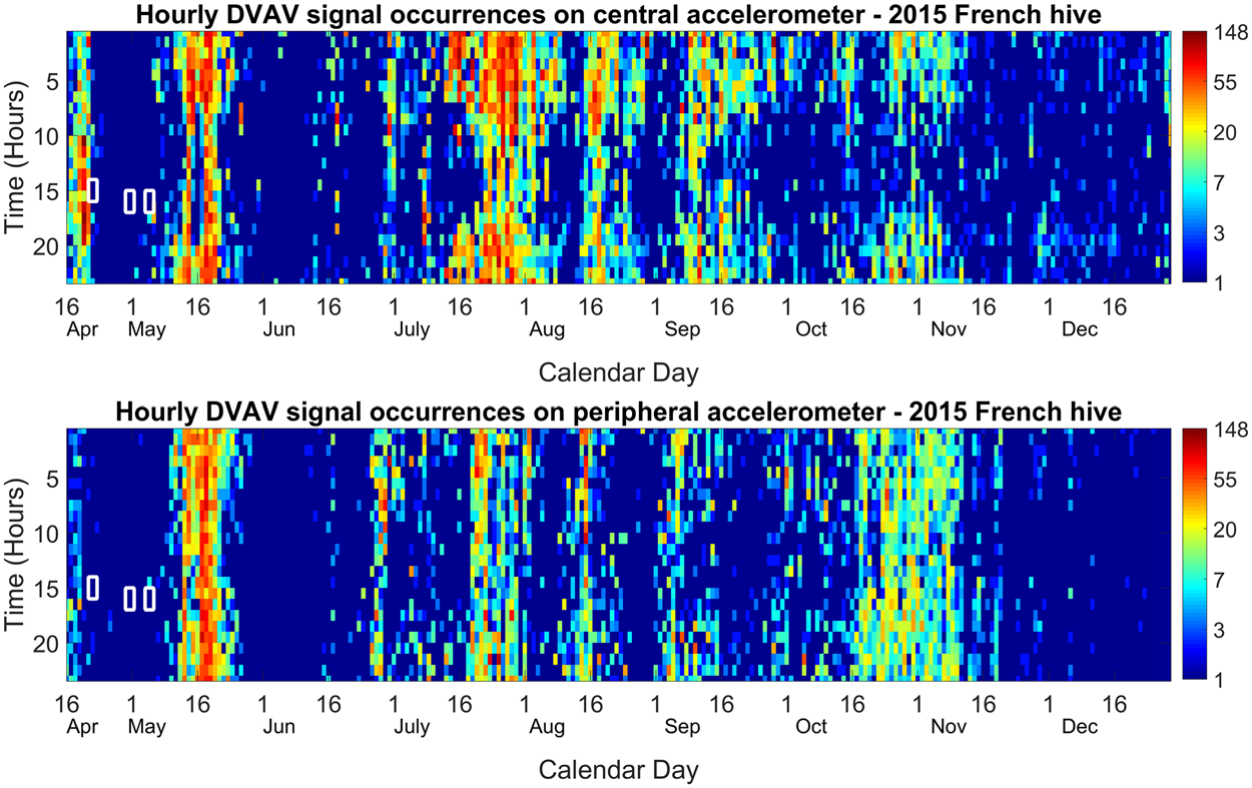
DVAV signal hourly occurrences. Central (top) and peripheral (bottom) accelerometer logs of the French colony (2015 season). The colour codes the number of hourly occurrences from dark blue (1) to dark brown (148 signals) on a logarithmic scale. White boxes highlight the occurrences of the three swarms that took place from this hive, with the first one being the primary swarm.

**Figure 5:**
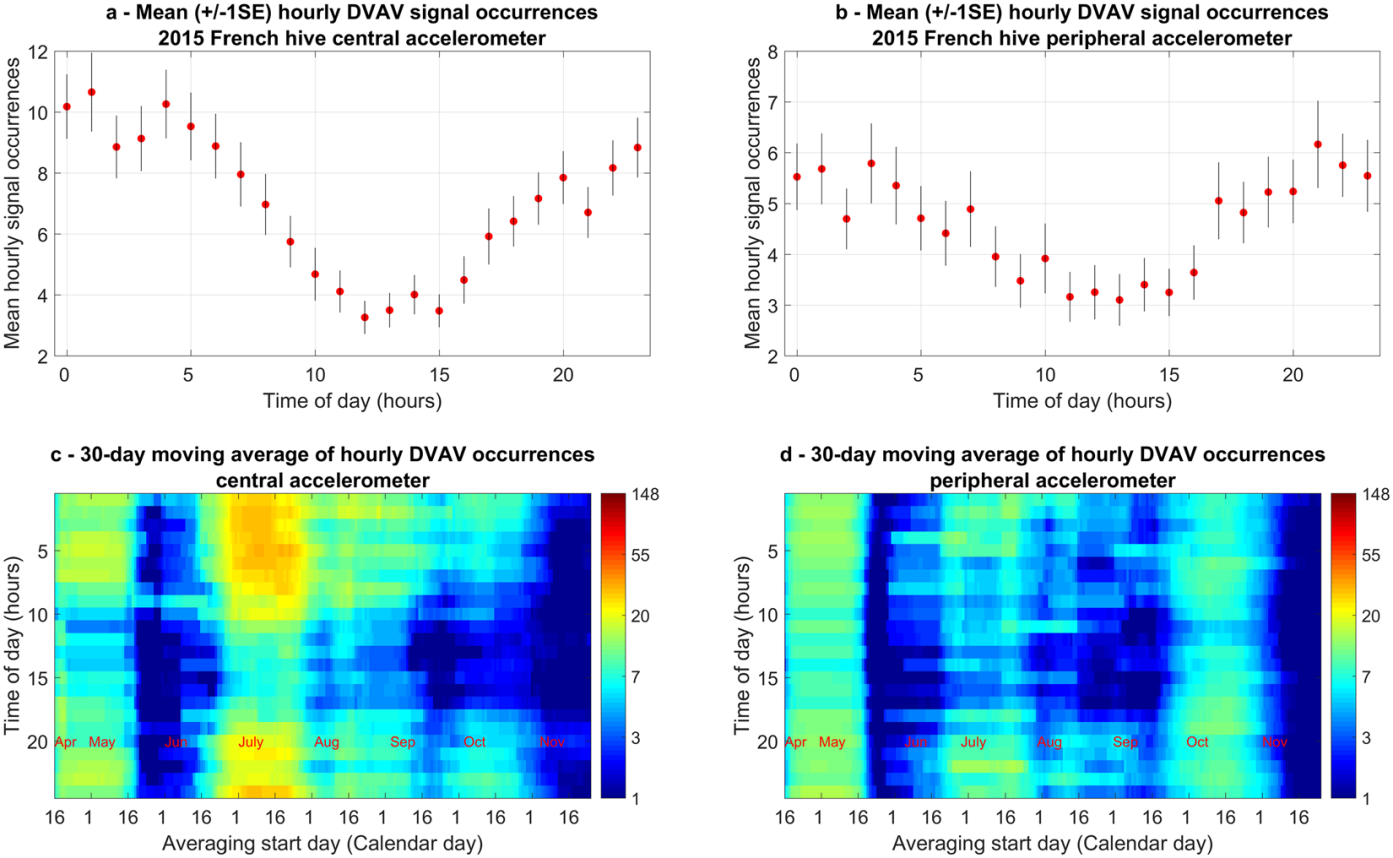
(**a**) The mean hourly trend in DVAV signals detected by our software over 24 hours across the entire dataset recorded on the central accelerometer; (**b**) on the peripheral accelerometer; (**c**) The mean hourly trend calculated over 30 dates and moved 1 day across the central accelerometer dataset; and (**d**) across the peripheral accelerometer dataset. This graph is obtained over the vibrational dataset shown in Fig. 4. The vertical bars indicate +/− 1 standard error (SE).

Figure 4 clearly demonstrates that the signal occurs relatively frequently within the immediate vicinity of the accelerometer, recorded at up to twice per minute with an average of nine per hour. There is also a pronounced and consistent decrease of occurrences at around 2 to 4 pm, further evident upon averaging DVAV signal occurrences for every hour across each day of the recording (Fig. 5a). Figure 5b also shows that this hourly trend holds stable across the entire dataset. This “lunchtime lull” is also apparent within the observation hive as well as the French 2017 hive (Figs S5 and S6a). It is seen that there is a large increase in DVAV signals detected in the days before and the hours immediately following the primary swarm, with a substantial decrease thereafter. Upon critical listening to these detections, audible DVAV signals can only be heard on the central accelerometer, and at 55 audible pulses an hour, there is no other point within the entire recording where strong DVAV signals can be heard in such high density. There appears to be a good correlation, over the entire season, between the two accelerometers, a trend that was not observed in the detection of whooping signals in Ramsey *et al*.^48^. Our dataset also reveals a high occurrence of night-time (between 8 pm and 5 am) DVAV signals, never shown in any previous study. There is a significant positive correlation between the modal midnight amplitude of^47^ the entire dataset and the daily mean number of DVAV signals recorded by our software on both the central (Supplementary Fig. S7) (*R*_*s*_ = *0*.*2475*, *p* < *0*.*001*) and peripheral (Supplementary Fig. S8) (*R*_*s*_ = *0*.*3505*, *p* < *0*.*001*) accelerometers, with peaks every 21–24 days. Interestingly, there is no correlation between the hourly number of DVAV and whooping signals^48^, recorded on the central (Supplementary Fig. S7) (*R*_*s*_ = *0*.*0735*, *p* = *0*.*2461*) or peripheral (*R*_*s*_ = *0*.*256*, *p* = *0*.*1002*) accelerometer (Supplementary Fig. S8). Finally, there appears to be a gradual reduction in overall signal production captured by the accelerometer as the year progresses into the winter with a sustained drop-off from early November. Amongst other signals, including worker pipes, wax scratching and wax chewing, (that were successfully discriminated by our software), it is easy to check (through computation of the individual pulse’s 2D-FT) the validity of the DVAV signals highlighted by red hotspots. Through the visual inspection of the 2D-FT images computed for 607 detected signals chosen at random throughout the year, we estimated the reliability for our detection software: 83% of the pulses elicited vertical broad bands at horizontal frequencies previously demonstrated to be unique to the DVAV signal. An audio file containing a collection of audible DVAV signals concatenated using the timings around midnight on the 18^th^ April is provided in Audio S1 for the listener to appreciate the high degree of repeatability of this unique type of honeybee signal.

In Fig. 5a,b, the hourly number of DVAV signals that have been detected by our software within the French 2015 dataset for each hour of the day was calculated and averaged over all days across the dataset for both accelerometers. It can be seen that there is a maximum between 11 pm and 4 am and a minimum between 11 am and 4 pm with smooth gradual increases and decreases in between. This trend is generic as it can also be observed on the peripheral accelerometer (Supplementary Fig. S10), across the Clifton observation hive (Supplementary Fig. S9a,c) and the French 2017 hive (Supplementary Fig. S6b) and is supported by video analysis in Table 2.

In Fig. 5b and d, the average number of DVAV signals for each hour of the day is only calculated over a 30 day period that is shifted along the time axis in one day increments (“moving average”). These show that the hourly trend seen in Fig. 5a and b is stable and holds across the entire active season. The same trend can be found in the Clifton observation hive (Supplementary Fig. S9b,d) and the French 2017 hive (Supplementary Fig. S6c).

**Figure 6:**
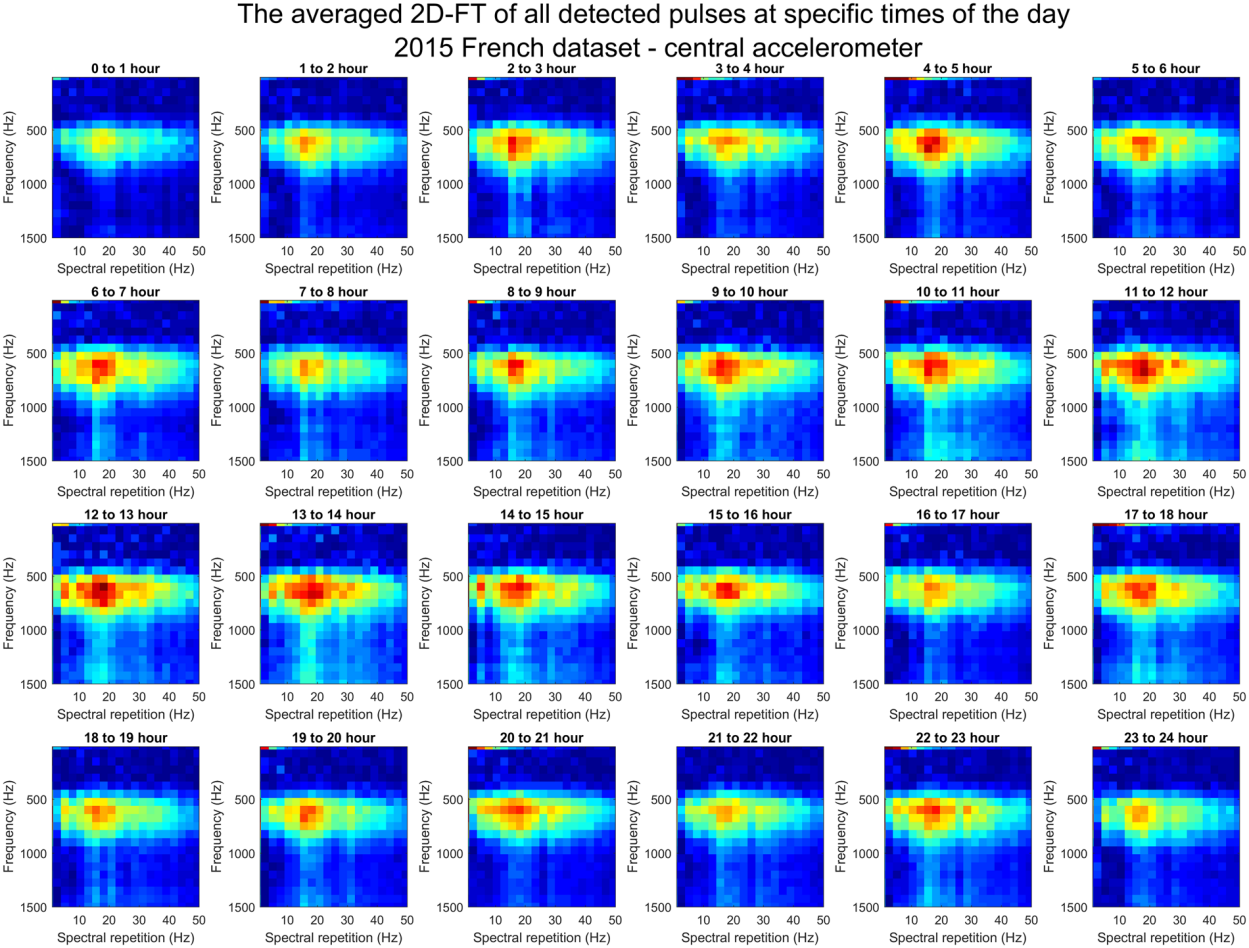
The mean 2D-FT of all DVAV signals detected on the central accelerometer by our software for each hour of the day throughout the full 2015 French dataset. Each plot is in arbitrary units with pixel intensity showing the amplitude of the acceleration from highest (deep red) to lowest (dark blue).

**Figure 7:**
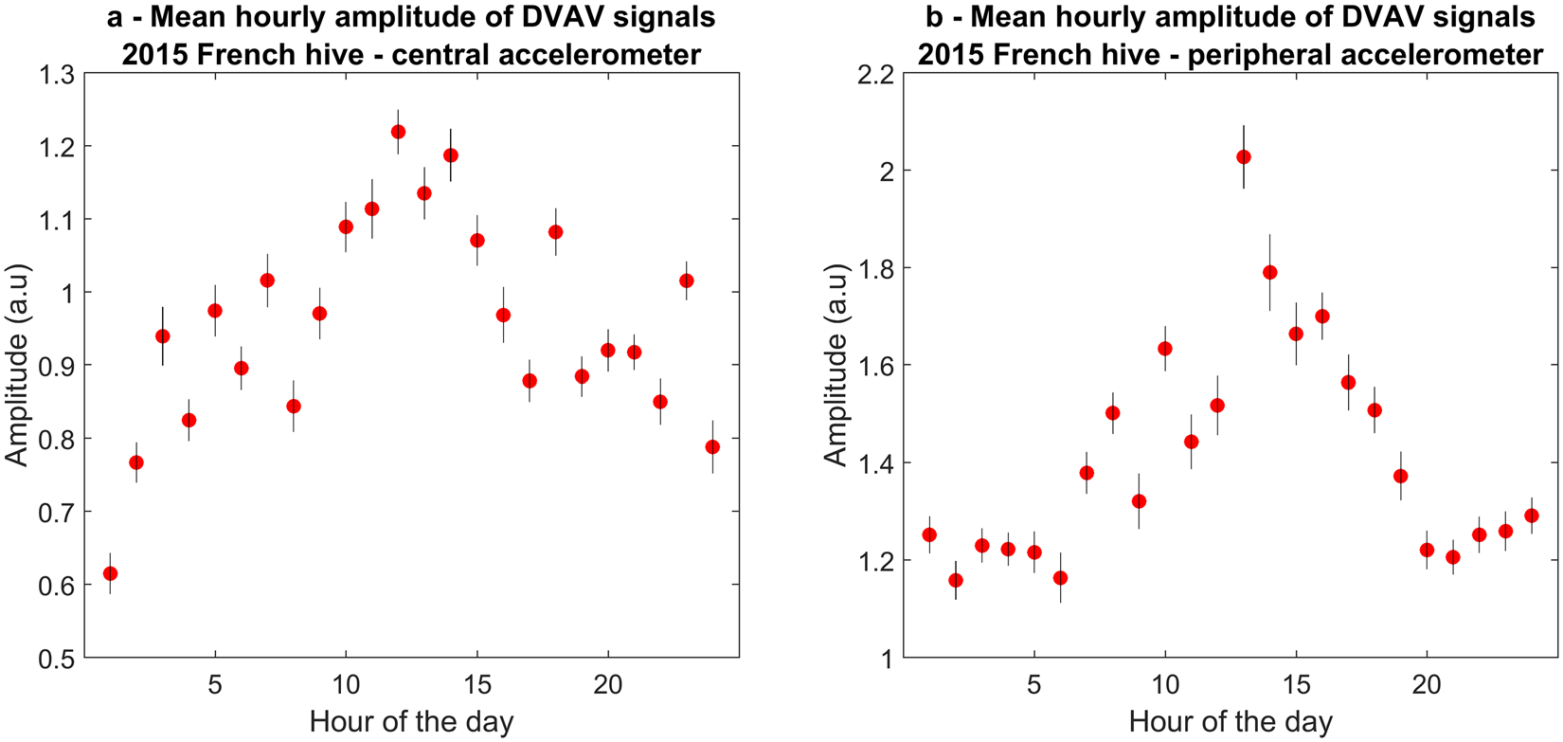
The mean (±1SE) hourly amplitude for the 14–25 Hz horizontal spectral bandwidth of the DVAV signals detected within the 2015 French hive dataset on (**a**, left) the central accelerometer and (**b**, right) the peripheral accelerometer.

To investigate any possible change in DVAV signal features across the day, all DVAV signals that were detected across the entire 2015 French dataset were categorised into one of twenty four groups depending on which hour of the day it was detected. The mean of the 2D-FTs over all pulses within each group was calculated and displayed in Fig. 6 for detections coming from the central accelerometer; see Supplementary Fig. S10 for the peripheral accelerometer data of the 2015 French dataset. It must be noted that the signal to noise ratio of these DVAV signals was so low that in order to display the 2D-FT image of the DVAV signal, the background noise had to be removed from the pulsed vibration by subtraction of data immediately following the pulsed vibration as in Ramsey, *et al*.^48^. It can be seen that there is no effect of the time of day on the spectral repetition of the broadband frequency spectrum associated with the DVAV signal, with it stably centred around 14–25 Hz across all hours of the day, a trend that is also seen on the peripheral accelerometer data (Supplementary Fig. S10), the Clifton Observation Hive data (Supplementary Fig. S11, left and Supplementary Fig. S12, right) and the 2017 French data (Supplementary Fig. S13). However, a pronounced trend can be seen in the amplitude of the signal, and this is further emphasised in Fig. 7, where it is seen that the amplitude of the mean 2D-FT for each hour increases steadily from midnight, peaks at 2 pm and decreases again until 11 pm. This trend is also seen in the 2017 French data (Supplementary Fig. S14), and is most apparent in the Clifton Observation Hive data (Supplementary Fig. S15a for the left accelerometer and Fig. S15b for the right accelerometer). Plotted in Fig. 7a is the mean amplitude of the 12–25 Hz bandwidth component of each mean 2D-FT from Fig. 6. Interestingly, the trend appears inverse to that of the hourly occurrence of DVAV signals (Fig. 7a,b), with higher amplitude DVAV signals occurring at times when the overall number of detections exhibits a minimum. Spearman’s rank correlation coefficient confirmed that there is a strong negative relationship between the hourly number of DVAV signals and the amplitude of the detected signals for the central accelerometer *(rho* = *−0*.*703*, *p* < *0*.*001)* and for the peripheral accelerometer *(rho* = *−0*.*835*, *p* < *0*.*001)*. This trend is also observed for the Clifton Observation hive left accelerometer *(rho* = *−0*.*896*, *p* < *0*.*001)* and the right accelerometer *(rho* = *−0*.*668*, *p* = *0*.*0155)* and the French 2017 hive dataset *(rho* = *−0*.*898*, *p* < *0*.*001)*.

Interestingly, the DVAV signals that were detected at the periphery had a 50% higher amplitude overall than those found at the centre of the frame (*t* = *8*.*308*, *p* < *0*.*001*), with a mean amplitude of 1.453 ± 0.252 (a.u.) compared to 0.947 ± 0.146 (a.u.), respectively. There is no significant difference between the amplitudes of DVAV signals detected by the left (mean = 0.7817 ± 0.136; Supplementary Fig. S12) or the right (mean = 0.7917 ± 0.321; Supplementary Fig. S13) accelerometers that made the Clifton observation hive dataset that were placed on the same horizontal axis equidistant from each other and the wooden frame *(t* = *−1*.*35*, *p* = *0*.*893)*.

The amplitude of DVAV signals that occurred over each hour of recording was computed and is displayed over each day, in Supplementary Fig. S16 for the central accelerometer and Fig. S18 for the peripheral one, which allows us to investigate this hourly phenomena on a daily basis. However, as seen in those figures, it is difficult to observe any trend over a single day. It is only upon averaging the data across enough days (e.g. 20, Supplementary Figs S16b and S18b), that the trend in Fig. 6 and Supplementary Fig. S10 becomes more apparent.

Cumulating the amplitudes of the DVAV signals detected for each hour (Supplementary Figs S17 and S19), rather than averaging, allows us to highlight instances of DVAV signals that are both numerous *and* strong. At 11 am on the 20^th^ April 2015, the highest sustained cumulative amplitude of our entire recording can be observed, and this is followed by the hours either side of the primary swarm, confirming that these sections of recording contain the highest density of high SNR DVAV signals (>50 per hour). In the hour containing the primary swarm, there are no DVAV signals detected and thus the cumulative signal amplitude is low. When summating the hourly amplitudes (Supplementary Figs S17a and S19a) of DVAV signals, the opposite trend can be observed compared to the computation of the mean (Supplementary Figs S16b and S18b), due to more DVAV signals having been detected at night (around three times more), as compared to the middle of the day (Fig. 4).

**Figure 8:**
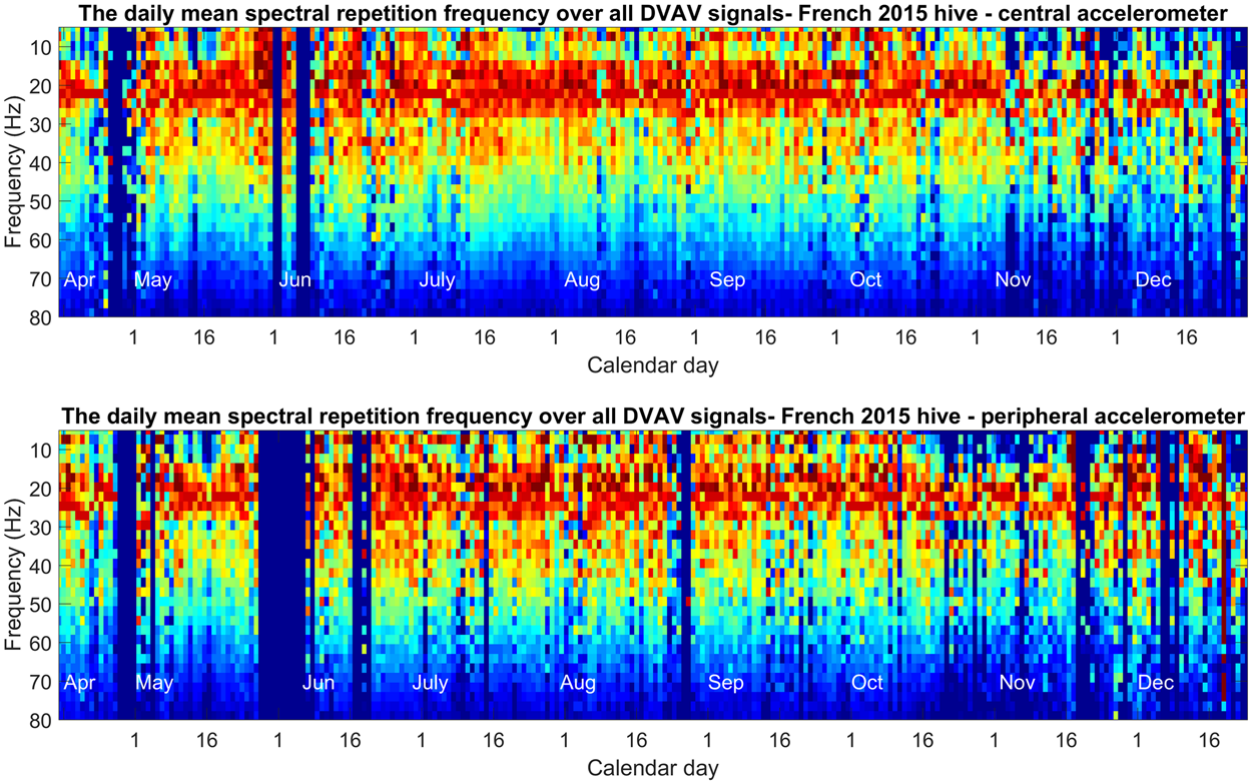
The daily mean spectral repetition frequencies of the DVAV signals detected within the 2015 French hive dataset for the central accelerometer (top) and the peripheral accelerometer (bottom). The x-axis shows the calendar day over which the 2D-FT average was calculated, the y-axis is the spectral repetition frequency of the 2D-FT image and the pixel intensity shows the amplitude of the acceleration of the DVAV signal in arbitrary units, scaled to its maximum every day.

In Fig. 8, we see that the peak frequency of recorded DVAV signals remains stable across the year at a fundamental frequency of between 14 Hz and 25 Hz. There is a reoccurring strong peak at 7 Hz that disappears in the winter time. Further, the mean peak frequency over the year is 20.299 Hz ± 2.95, supporting the findings of Fig. 2. This trend is confirmed by simple linear regression that deduced that the peak frequency of detected DVAV signals cannot be predicted by the day number (*r* = *0*.*0011*) with data showing statistical significance (*p* < *0*.*001*). The same trend can be seen for the peripheral accelerometer displayed in Fig. 8b, with no relationship occurring between frequency and time (*r* = *0*.*024*, *p* = *0*.*283*).

As seen for the French 2015 hive (Fig. 8), the data pertaining to the mean daily 2D-FT of the Clifton Observation Hive (Supplementary Fig. S20) shows that the frequency of the DVAV signals also remains stable across this recording and is centred at a mean of 19.679 Hz ± 3.21. This again is confirmed by simple linear regression showing that the peak frequency of detected DVAV signals cannot be predicted by the day number for the left *(Frequency: r* = *0*.*054*, *p* = *0*.*0269)* or the right accelerometer *(Frequency: r* = *0*.*035*, *p* = *0*.*149)*.

This stability in frequency is also apparent for the French 2017 dataset hive (Supplementary Fig. S21) across the entire active season until the colony collapsed in December *(r* = *0*.*022*, *p* = *0*.*012)*. The data becomes more scattered during the final months of the recording as signal detection becomes rarer. The DVAV signals in this dataset are centred at a mean of 20.101 Hz ± 3.21.

### DVAV signals and weather

**Figure 9:**
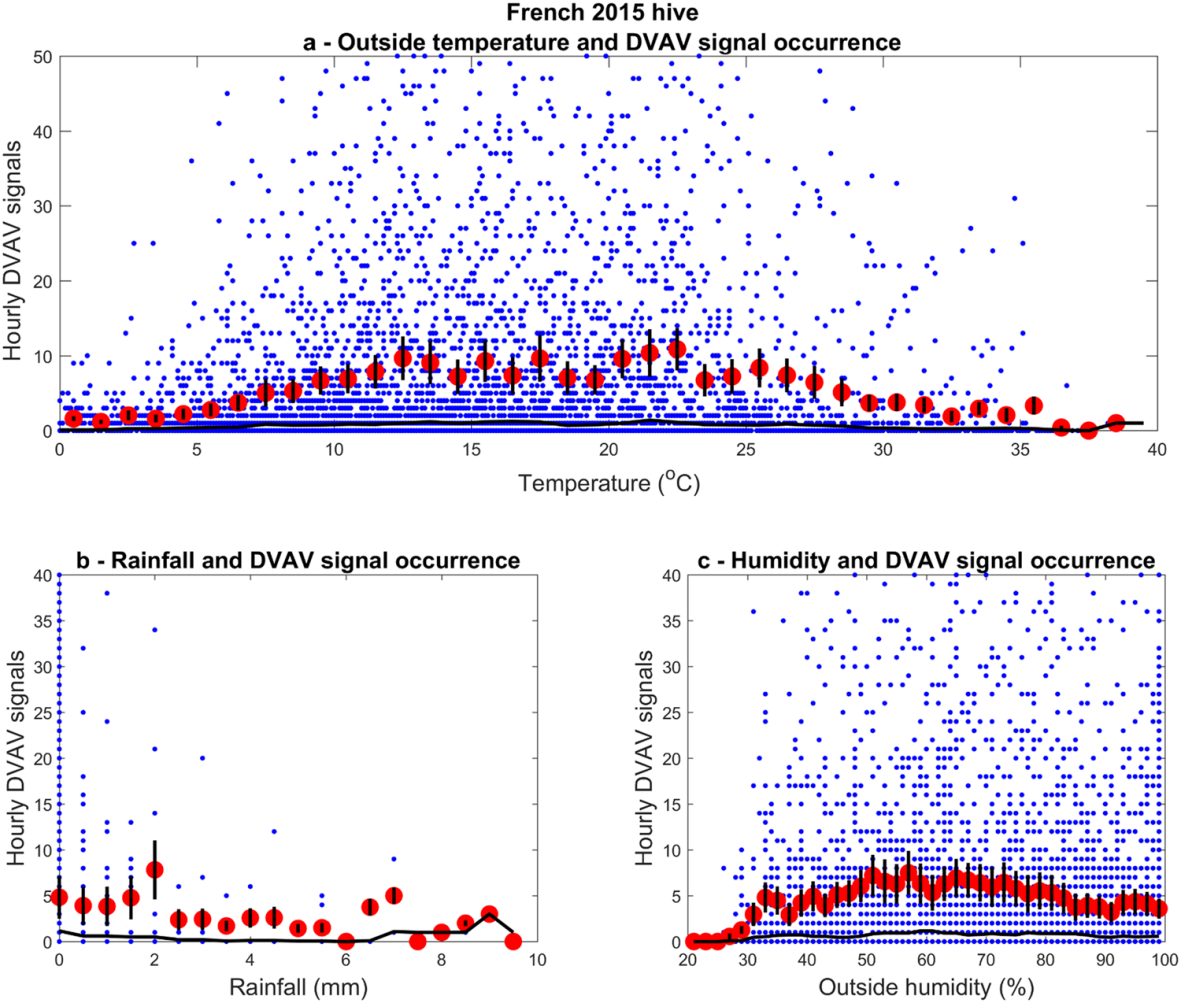
Hourly occurrences of DVAV signals in relation to weather. French dataset (2015 season) with corresponding: (**a**) average outside temperature, (**b**) cumulative rainfall, and (**c**) average outside humidity. Red dots indicate the average number of DVAV signals with black bars displaying ± 1 SE. The black curve on each graph shows the modal hourly DVAV signals.

Plots of the complete hourly data for temperature, humidity and rainfall that coinside with our 2015 recordings can be found in Supplementary Fig. S22. It can be seen in Fig. 9a that the occurrence of honeybee DVAV signals is at its lowest at the extremes, i.e. at 0 and 38 °C, a trend that is mirrored for the 2017 French hive dataset in Supplementary Fig. S23a. There is a steady increase in the occurrence of DVAV signals from 0 to 12 °C and then a plateau until 27 °C when a steady decline can be seen thereafter. The same trend is displayed in the humidity plot in Fig. 9c, where a steady increase in DVAV signal occurrences can be seen until around 40%, with a steady decrease after 80%. As seen for the 2017 French hive dataset in Supplementary Fig. S23b, there is also no perceivable trend between rainfall and the number of DVAV signals but it can also be seen that the majority of days saw very little precipitation. However, one day of prolonged heavy rain on the 28^th^ October 2015 (Supplementary Fig. S22a) appears to correspond to a day clearly lacking of the late afternoon lull in the occurrences of DVAV signals (Fig. 5), in steep contrast with the rest of the data.

### Supporting video evidence

For all videos comprising of a soundtrack, it is made strictly from the raw data from the accelerometers embedded in the honeycomb under video analysis. The left channel always corresponds to the left accelerometer of the side with visible sensors, if the video camera is facing the other side of the frame then the channels are reversed.

A honeybee delivering a DVAV signal directly onto one embedded accelerometer can be seen and heard in Video S3. For comparison, a honeybee delivering a DVAV signal on the other face of the frame, near the same embedded accelerometer (not visible), can be found in video S4. Both produce high SNR traces. In video S4, following the first audible DVAV signal, the signaller is seen to move slightly away (~5 mm) from the accelerometer and produces a second, inaudible DVAV signal. This demonstrates how local the DVAV must be to the accelerometer in order to be audible and it also provides evidence that we are able to detect DVAV signals on both sides of the frame.

Both DVAV signals display the characteristic Π-shape in the frame by frame image of the time course of acceleration also shown in synchrony in Videos S2 and S4. The major difference between the videos is that each knock for the DVAV performed on top of the accelerometer (Video S3) results in a sharp negative burst of acceleration whereas in Video S4, it is positive. This provides evidence that the polarity of the individual knocks that make up a DVAV signal is disclosing the side of the frame on which the signaller is residing.

In Videos S2 and S4 we also show the instantaneous 2DFT computed in synchrony with each of the DVAV signals that occurred on each side of the frame. On both videos, two vertical bands at around 17 Hz and 34 Hz can be observed. Furthermore in Video S4, as the bee moves further away and produces an inaudible DVAV signal, the vertical bands still appear on the 2DFT image showing how sensitive and useful the 2DFT is in the analysis of these pulsed vibrations that convey very weak audible signals.

Provided in Video S5 is the analysis of the collection of 27 high-SNR pulsed vibrations that were used in the initial vibrational quantitation of DVAV signals. The time course of the acceleration, the image of the time course chopped into its individual knocks, the mean acceleration of a typical knock and the 2D-FT image are all provided, together with the raw accelerometer recording in the audio of the movie. It can be seen that each elicits the characteristic Π-shape in the knocks, as the signaller’s rate of delivery increases, remains constant and finally decreases. These knocks can be seen to have either sharp positive or negative acceleration depending on the side of the accelerometer the DVAV signal was performed. Analysis of the 2D-FT for each signal showed that each systematically elicited two vertical broad bands at a spectral repetition corresponding to that of the number of knocks (between 13 and 24 Hz), and a second harmonic at twice higher frequency. These DVAV signals have such high SNR that no background removal is necessary for their analysis.

To further demonstrate the sensitivity of 2D-FT analysis for DVAV signal examination, in Video S6 we present an example of an inaudible DVAV signal that was successfully detected by our software. We show that the vertical bands on the 2D-FT image that are associated with honeybee DVAV signals are clearly visible even though no audible information can be perceived. This, in combination with other examples of inaudible true detections (Supplementary Table S1), is strong evidence that the 2D-FT is a better assessment of the validity of the accelerometer trace that pertains to DVAV signals than human hearing.

## Discussion

The DVAV signal is well known from visual inspections of honeybee activities upon the comb, requiring an observation hive and necessarily resulting in relatively short-term measurements. However, this work discloses a new method of measurement of DVAV signals that does not rely on any visual inspection, works on time durations longer than that of the colony’s lifespan, gives detailed quantitative assessment of each DVAV signal, reveals the side of the frame where the signaller resides, does not require illumination, frame extraction or specialised hive design, and works at any time of the day.

Our initial analysis of the physical properties of DVAV signals is in-keeping with the results of past previous researchers, such as Gahl^24^. Owing to the repeating pattern of individual knocks that make up the DVAV signal, we show that the existence of these features within a signal waveform can easily be identified using 2D-FT analysis, even when the signal to noise ratio is exceptionally low. Through our work, the 2D-FT can therefore be considered as a tool more powerful than critical listening for detecting and assessing the validity of detected DVAV signals, where strong vertical broad bands at narrow horizontal frequencies around 20 Hz and multiples, known to be associated with DVAV signals, are clearly seen.

In this study, we show for the first time that there is an unprecedented increase in the cumulative amplitude of DVAV signals in the hours preceding and following a primary swarm. These, however, can only be heard on one channel, but the occurrence plots (Fig. 4) exhibit enhanced DVAV signals both on the peripheral and central accelerometers. As we have shown that the spatial distribution of DVAV signals is even across the comb, this provides further evidence that our algorithm detects DVAV signals that are not necessarily audible.

DVAV signals at night were found to be lower in amplitude but higher in occurrence, and those detected during the “lunchtime lull” had a higher amplitude. One possible explanation for this is that those DVAV signals that take place during the day may be associated with excited foragers returning from resource patches conveying urgent information. In contrast, at night time the individuals are less frantic within the hive as the urgency of foraging has diminished. An important remaining question is why is there an activation signal occurring so commonly at night time. Perhaps there is a function beyond that of being solely a honeybee activator. If the purpose of the DVAV signal differs at night time, then this would also suggest that the function of the DVAV signal can be switched by the amplitude that the signal is delivered with. It is also possible that at night there is a shift in the ratio of DVAV signals in favour towards those that are delivered directly onto the honeycomb. This night time enhancement in the occurrences of the DVAV signal may be consistent with existing proposed functions. The level of signalling activity has been shown by Schneider *et al*.^16^ to correlate with foraging success and the resulting increased food intake could facilitate increases in many in-hive activities such as food processing and brood care. The DVAV signal has been demonstrated to enhance the performance of these tasks that are undertaken continuously by in-hive workers that experience little or no circadian rhythm^49^. The high rates of DVAV signalling observed throughout the night may help to maintain a level of brood care coordinated with the influx of food during the preceding daylight hours. Finally, night time DVAV signalling could help to maintain a certain level of arousal in the foraging caste to facilitate the rapid reactivation of foraging the following morning. This is in line with Schneider *et al*.^16^, who also observed a decrease in the amount of DVAV signalling behaviour throughout the day, suggesting that the increased morning DVAV signalling may exert a priming influence on foraging behaviour.

Observations throughout this study suggested that the abdomen of a honeybee performing a DVAV signal made contact with the honeycomb around 30% of the time, regardless of the intended recipient. It has also been shown that the honeycomb responds vibrationally in a different way depending on the load within the frame, further supported by the significant increase observed between the amplitude of DVAV signals detected at the bottom periphery of the frame compared to those at the centre, and the results of Sandeman *et al*.^50^. It could therefore be that honeybees can use the comb mechanical response of the DVAV signal to make assessments into the local contents of the frame below them, by producing this signal directly onto the honeycomb then sensing and analysing the vibrational response. At night, the probability of a honeybee delivering a DVAV signal into the honeycomb might be much greater as they assess the honeycomb beneath for the storage of the days forage. Additionally, this variation in honeycomb frame-load could also be the driving factor behind the positive correlation observed between the number of detected DVAV signals and the brood cycle, monitored on the focal frame in terms of the daily modal night time overall signal amplitude^47^. The developing brood causes a steady increase in the honeycomb’s density as they grow within its cells, until they hatch, when the density becomes suddenly much lower. This could cause a reduction in the detection range of the accelerometer that intensified over the 21-days of development and the 21-day peaks in Fig. 4. Using several accelerometers it is possible to sense the brood in all frames of a hive^47^, and this could allow future work to check the previously observed positive correlation between the DVAV signalling and the extent of the brood (Hyland 2007).

For accelerometer technology to be an effective method of sampling DVAV signal occurrences, the signals must therefore appear to be delivered homogenously across the honeycomb, even when the density of individuals is low and the percentage of DVAVs delivered onto the honeycomb must be constant when compared to those delivered onto another honeybee. The fact that the number of detected DVAV signals correlates positively between the two accelerometers in Fig. 4, for the French 2015 hive, and Supplementary Fig. S5 for the Clifton Observation hive, is in keeping with the results of Fig. 3, which suggests that regardless of the number of DVAV signals that occur the distribution should be even across the honeycomb, further supporting that the use of just one accelerometer placed in the centre of the honeycomb is sufficient in capturing a meaningful and representative general view of the number of DVAV signals occurring at that time on that frame, providing that the accelerometer measurement is long enough. Thorough observations of video data suggests an hourly average of 339 DVAV signals on this particular frame. Therefore, our data showing that we capture an average of nine per hour on the accelerometer suggests that perhaps 2% of the DVAV signals delivered on the frame are captured by this method, however this has shown to be as high as 30% at times of low frame-load. This is to be expected as we were only focused on DVAV signals that occur (i) directly on the honeycomb and (ii) on or in the immediate vicinity of our sensors thus we would suggest that we were only sampling around 2% of the frame’s total surface area. Due to the homogeneity of DVAV signal delivery across the honeycomb (Fig. 3), sampling such a small surface area can be still be meaningful.

The DVAV correlation plots with weather are remarkably scattered, similar to that seen for whooping signals^48^.The detected DVAV signals are shown to be most frequent between 13 and 28 °C. This is most probably a seasonal effect, with more DVAV signals being detected during the active season than during the colder winter months. The fact that there appears to be an upper threshold for temperature, where honeybees start producing less DVAV signals further supports it being a signal associated with foraging, as bees are known to restrict foraging above and below certain outside temperatures^51^. In keeping with the results of this research, other studies have shown that outside temperatures around 20 °C result in the highest level of foraging from focal hives^52^, while temperatures exceeding 40 °C^53^ and preceding 10 °C^54^ result in the lowest observation of foraging activity.

Shown in the hourly weather plots in Supplementary Figs S22 and S24, there is a negative relationship between outside humidity and outside temperature. Therefore, the relationship between outside humidity and DVAV signals was expected to be inverse of that between DVAV signals and temperature. Nevertheless, the same effect of outside temperature can be seen for outside humidity in the weather analysis of the French 2015 data. However, in the French 2017 weather analysis, whilst the same effect of temperature on DVAV signals can be seen, there appears to be no trend associated with humidity. This suggests that humidity and temperature act upon DVAV signals independently and the effect of humidity is perhaps driven by diurnal variations. As seen in Supplementary Figs S22 and S24, the humidity is highest during the night, when most DVAV signals are detected. However, the reduction seen above 80% humidity can be attributed to the decrease in DVAV signals detected in the winter time when humidity reached its peak in the 2015 season. It therefore seems unlikely that humidity has any great effect on the occurrences of DVAV signals on its own, supporting the findings of Joshi and Joshi^54^.

Schneider, *et al*.^45^ created artificial rainstorms to trap foragers inside the hive for consecutive days and found that over time this reduced the colonies DVAV signalling activity. In this study, however, we found that rainfall had little effect on the occurrences of DVAV signals with one day of prolonged heavy rain (28^th^ October 2015) coinciding with an enhancement in signal detection. As seen for the French 2015 weather data (see Supplementary Fig. S22a) and French 2017 weather data (see Supplementary Fig. S24a), days of prolonged and heavy precipitation are actually quite rare, skewing the analysis, and preventing us from making meaningful comparisons with Schneider’s rainstorm experiments. This could explain why, overall, rainfall had little effect on DVAV signal occurrences. However, it can also be explained by the dual-functionality of DVAV signals proposed by this research. It is widely known that honeybees do not forage in the rain returning to the hive to take shelter. It is therefore to be expected that a foraging signal would cease during these times. The overall lack of trend between rainfall and the occurrence of DVAV signals is another example (in addition to the increased occurrences of DVAV signals at night) where DVAV signals occur outside the remit of foraging.

In keeping with Painter-Kurt and Schneider^26^, our study also supports the hypothesis that DVAV signals are predominantly a product of the foraging caste. Firstly, they tend to occur more at times when the foragers would be present in the hive (in the mornings, the evenings and during times of heavy rain). Secondly, our daily histogram of occurrences also fits with Nieh’s^22^ findings that foragers tend to produce more DVAV signals in the mornings prior to foraging flights and in the evenings when they return to the hive. It also agrees with the interpretation “prepare for greater activity”^22,23,37^ as in the evenings the foragers would be returning from a day’s forage causing a sudden surge in nectar and pollen influx. The reduction of DVAV signals during the winter months, a time when the amount of available resources for the bees will be minimal, is further suggestive that this signal may be linked to the foraging caste.

Our peaks in DVAV signal detection throughout the summer months of recording are also in-keeping with observations of Schneider *et al*.^16^. In their study, autumn and winter months exhibited smaller and less frequent morning DVAV signal peaks, and these tended to coincide with, rather than precede, foraging activity. On the daily histogram, we also see an enhanced number of DVAV signals that are detected prior to the primary swarm (further supporting the “prepare for greater activity” hypothesis) that is followed by a sudden drop off in the subsequent hours, only returning in the hours after the final swarm takes place. It is possible that this is related to the average age and the number of foragers that are remaining within the hive. Until the swarming season is over, the average age of the colony’s population is much lower as the majority of the older foragers left with the primary swarm. It may also be the case that the majority of DVAV signals are localised to the cells of developing virgin queens during this period^28,29,37^. Future work will involve further assessment of the vibrational properties of the DVAV signals extracted from vibrational datasets of honeybee colonies that are experiencing adverse conditions, such as intoxication from pesticides. It is possible that colonies experiencing specific health disorders do exhibit detectable variations in the characteristics of DVAV signals and due to the remarkable stability of the features that have been shown in this study, any deviations from normality should be easy to spot. One way to confidently make this assessment would be to monitor declining colonies in observation hives, linking the DVAV signals that were produced to their exact accelerometer waveforms. If this could be achieved, it would provide an effective early warning sign for beekeepers. It would also be beneficial to assess this signal in relation to agricultural practices. For example, it would be interesting to examine the effect that the sudden harvesting of flowering crops such as oil seed rape (*Brassica napus*) has on the DVAV signal production. This crop is planted in spring and harvested in the early summer. During this time, local honeybees have an abundant forage source and the honeybee colonies massively expand as a result^55^. Upon mass harvesting in early summer it is expected that these large colonies will have lost their primary foraging resource at the peak of their active season. It is therefore to be expected that this signal would significantly reduce after the crop harvesting, as this reduces massively the availability of forage. However, peaks in DVAV signal production will occur as new foraging patches are found^23^.

To conclude, we present here a study that has made significant advancements in the use of accelerometer technology to study honeybee mechanical communication without the severe limitations imposed by visual observations. Through use of analysis techniques that are novel to the science of animal communication we have successfully been able to quantitate the physical properties of a signal that was thought to have no vibrational component, presenting strong evidence for the one-to-one relationship between the signal and an accelerometer trace, and this has led to a unique signature that has provided us with the capability to study this signal continuously within long term vibrational datasets. It is through this long term analysis that we realise that there is still much more work needed to decode the meaning of this signal and to what extent it can be used as a proxy for colony health. The present results add another category of honeybee vibrational pulse, the DVAV signal, to the collection of pulses that we already can successfully detect^48^ (whooping signals, worker pipes, queen toots and queen quacks), bringing us closer to our long term goal in which all categories will be automatically logged, without the need to store the raw data, providing a sensitive tool for the non-invasive assessment of honeybee colony status.

## Methods

No ethical approval was required as this study wholly focussed on the *in-situ*, non-invasive acquisition of data from colonies of invertebrates. There are also no competing interests associated with this work.

### Continuous Recording of vibrational data

The configuration of the hardware involved for the continuous recording of the vibrational data set was identical to that used by Ramsey *et al*.^48^ for the detection of honeybee whooping signals. The accelerometers in this study were calibrated using an Aim-TTi TG5011A 50 MHz Function Generator to drive 50 mV directly into our sound card.

Three hives were monitored as part of this study. The first named “French 2015 hive” is the same vibrational dataset as used for the “French hive” in Ramsey *et al*.^48^, and was a Dadant beehive consisting of a ten-frame brood box and ten frame honey super continuously monitored from 16^th^ April 2015 until 26^th^ December 2015 with two accelerometers vertically aligned on the central brood frame, disclosing the evolution of a colony across the entire active season. This colony swarmed three times within the first month of the dataset.

The second hive is named the “Clifton Observation hive” as it was located on the Clifton campus of Nottingham Trent University, UK. This hive was used to collect all of the video recordings as part of this study, and is a custom made observation hive consisting of two 10-frame British National brood boxes. It was continuously monitored (and is still monitored today), as for the other hives, starting on the 24^th^ May 2016 with two accelerometers horizontally aligned both equidistant from the surrounding wooden frame. This frame was at the periphery of the colony until it was placed at the centre after April 2017. This colony was also found to have superseded their queen in June 2017.

Finally, the “French 2017” hive comprises of the same hardware as that of the 2015 season, both located at the same apiary in Jarnioux, France. It was monitored from 15^th^ April 2017 until 28^th^ November 2017, but using a single accelerometer placed at the centre of the middle frame of the colony. This colony did not swarm and died at the end of the 2017 active season.

### Video recordings

For the collection of video data, the same observation hive was used as in Ramsey *et al*.^48^. The camera arrangement was identical to that of Ramsey *et al*.^48^, with both cameras recording in 1080p definition at 50 fps. The higher framerate, at well above twice the frequency of a normal DVAV signal, was essential to account for Nyquist ghosting^56^. To allow the bees to experience as natural conditions as possible, no more than two 10 to 20 minute recordings were conducted each week. During all other times, the bees experienced the same conditions that they would within a normal dark honeybee hive. This allowed this colony and the observation frame to develop naturally without the major disturbances usually associated with observation hives with static frames, where bees are forced to live in a planar geometry. Some excerpts of these videos have been included to support some of the claims and aid in the discussion of the statistics of these signals generated as part of this study as accelerometer traces usually provide very poor (if any) audible evidence of DVAV signals (in great contrast to “whooping signals”^48^). Video recordings were therefore essential to validate many of our claims.

### Vibrational quantitation of DVAV signals

Using a home-built MATLAB® (The Mathworks, USA) program, the waveforms were extracted from the dataset. The acceleration of the waveform was calibrated into m/s^2^ and the time course was cropped around the signals for careful further examination.

A collection of 27 DVAV signal waveforms was extracted from various vibrational datasets in the UK and France, and were each given an ID based upon the time and date at which they occurred. The waveforms were each analysed by the methods above, and they were carefully studied to highlight features unique to DVAV signals that can be later utilised within a supervised clustering algorithm to best distinguish DVAVs from other pulsed signals in a long-term scan of a colony’s vibrational log.

### Visual detection

From visual and auditory inspection of our extensive collection of video recordings, it is apparent that detection of an audible DVAV signal trace is entirely restricted to those instances where honeybees deliver it in the immediate vicinity of the accelerometer (2–3 cm radius), and directly onto the honeycomb rather than onto a fellow honeybee. Using the video data obtained from the observation hive across the 2016 and 2017 seasons, a grid was drawn over the honeycomb within the footage (see Video S7) in order to facilitate the recording of the coordinates at which each DVAV signal occurred. These were then plotted along with the mean and the median of the collection of coordinates to show the distribution of DVAV signals occurring over the honeycomb. All of the videos used in this analysis of spatial co-ordinates were recorded between 11 am and 1 pm.

### Long-term automated scan

Software was written in MATLAB® and algorithms were optimised to detect and record the times at which DVAV signals occurred within the vibrational data sets (See Supplementary Methods for more information). The optimised DVAV signal detection is a two-stage discrimination process. In the first pass, the software uploads 2-minutes of data from the file, creates a 1-second long window that moves along the slab by one tenth of a second each time. The waveform within the window is discriminated into ‘DVAV signals’ and ‘non-DVAV signals’ by simple discriminant function analysis on 2D-FT (2-dimensional Fourier transform) of the waveform under scrutiny. In order to obtain this, the spectrogram of the wave form is first obtained (i.e. power spectra of short contiguous acceleration samples are stacked from left to right), and the power spectrum of each of its horizontal lines is further computed.

Analysis of the 2D-FT in combination with some critical listening to the pulses detected in this way revealed instances of single vibrational “knocks” being occasionally erroneously identified as a DVAV signal. In the second pass, the waveform detected as a DVAV signal is further verified using a second test optimised to check the periodicity of the knocks in addition to their individual waveforms, which are further exaggerated by calculating the gradient of the acceleration. Once the end of the slab is reached, the software moves on to the next two minutes until the entire dataset has been scanned. A scan of a month of recorded data typically requires around 12 hours of computer processing. When data sets consisting of the timings of the honeybee DVAV signals that occurred within each vibrational data set are identified, quantitative statistics of their occurrences over the entirety of datasets can be showcased.

The validity of the automated detections of DVAV signals was examined using the vibrational data associated with the entire collection of our video footage. The detection software was run on the corresponding audio files extracted from the raw accelerometer vibrational datasets. The list of detections were checked against the corresponding DVAV signals within the footage (see Supplementary Table S1). To further demonstrate the sensitivity of the 2D-FT for the detection of DVAV signals, the 2D-FT of a correctly detected DVAV signal that produced no audible trace has been given in Video S6. After this, 629 randomly selected DVAV signals were extracted from the raw data file and the 2D-FT was computed and displayed for each to ensure they produced vertical broad bands between 14 and 25 Hz.

Upon completion of the long-term scan and validation of the results, a histogram of hourly DVAV occurrences was computed for each day displayed vertically, with the day under investigation displayed on the horizontal axis. The logarithm of the number of hourly occurrences is denoted by the pixel intensity of the colour plot.

### Long-term signal parameter analysis

All data analysis, statistical and graphical, was undertaken in MATLAB® with occasional use of the statistics toolbox. All data was tested for normality using the Kolmogorov-Smirnov test and in some instances, normalisation is undertaken using Log_10_ transformation. When normal distribution could not be achieved, the non-parametric equivalent test was used. The signals were checked for any duplicate detections, as this would suggest that we had not detected a local DVAV but something of much higher amplitude occurring on the honeycomb. No duplications were ever detected because of the close proximity essential for their detection.

### The brood cycle and daily average

To see how the occurrences of DVAV signals changed throughout the course of an average day, the number of DVAV signals that occurred at each hour of the day is averaged and standard error is shown in Fig. 5 for the French data, and supplementary Figs S2 and S3 for the Clifton Observation hive and the 2017 French hive, respectively. To investigate any association between DVAV signal production and the honeybee brood cycle, on the focal frame, the daily modal amplitude of vibrations as a whole (shown by Bencsik, *et al*.^47^ to highlight the honeybee brood cycle) is explored for the French 2015 dataset. In addition, the daily average of DVAV signals is also compared to the daily average of whooping signals previously shown in Ramsey *et* *al*.^48^, and can be found in Supplementary Figs S7 and S8.

### Daily Averaged 2DFT

To assess any seasonal changes to the DVAV signal parameters, all DVAV signals detected by our software for each day were extracted from the raw dataset, allowing a 2 second window centred on the pulse timing. After this the 2D-FT was calculated for each DVAV signal, the background information was removed by subtracting the 2D-FT of two seconds of data immediately following the end of the focal DVAV signal, and then the average of all 2D-FT images was computed. This was repeated for all days within the dataset. The mean was then calculated to show the average signal at each value of the spectral repetition frequency of each day’s mean 2D-FT, which was then plotted vertically in a colour plot with each day stacked horizontally in chronological order to show the evolution of the peak DVAV signal spectral repetition across all days for all datasets.

### The effect of weather on the occurrences of DVAV signals

The French 2015 and 2017 weather data was kindly supplied free of charge by Météo France (www.metofrance.com) for the site and dates that we required. The relationship between outside temperature, outside humidity and rainfall, and the hourly occurrence of DVAV signals recorded by our accelerometers was explored using a regression model. For the analysis with rainfall, linear regression was used to assess whether increased rainfall had an effect on the number of DVAV signals.

The weather data is included in Supplementary Fig. S22 for the 2015 season and Fig. S24 for the 2017 season. For ease of use, this figure has been formatted to match that of the hourly histogram computed from our long-term trends of DVAV signal occurrences.

## Electronic supplementary material

Combined Supplementary Material

S1 AudioS1 Audio

S1 Video (DVAV HD Movie)

S2 Video (Shaking run)

S3 Video (DVAV at 42s)

S4 Video (DVA_with_zoom_Osc_2DFT otherside)

S5 Video (DVAV stats)

S6 Video (Flicker-Free Silent DVA with sound)

S7 Video (Video with grid)

S8 Video (Bee working in an empty cell)

## Data Availability

All relevant data are within the paper and its Supporting Information files. Numerous raw accelerometer data files (600 hours of the 2015 French dataset) have already been uploaded in support of a previous publication^48^, to the following: Folder 15: 10.6084/m9.figshare.4815157 Folder 14: 10.6084/m9.figshare.4793653 Folder 13: 10.6084/m9.figshare.4789822 Folder 12: 10.6084/m9.figshare.4772362 Folder 11: 10.6084/m9.figshare.4765705 Folder 10: 10.6084/m9.figshare.4758511.
