# Supplementary material for: Extensive Vibrational Characterisation and Long-Term Monitoring of Honeybee Dorso-Ventral Abdominal Vibration signals
Source: Sci Rep. 2018 Oct 1;8:14571. doi: 10.1038/s41598-018-32931-z (PMC6167329; doi:10.1038/s41598-018-32931-z)
Supplement: Supplementary file 1 — Combined Supplementary Material [file 41598_2018_32931_MOESM1_ESM.pdf]

Nottingham Trent University

Extensive Vibrational Characterisation and Long-Term Monitoring of Honeybee Dorso-Ventral Abdominal Vibration signals

Supplementary Material

Ramsey M.[1], Bencsik M.[1] and Newton M.I.[1]

31st July 2018

[1] Nottingham Trent University, School of Science and Technology, Clifton Lane, Clifton, Nottingham, NG11 8NS.

Supplementary Media

S1 audio = Collection of high SNR DVAV signals from a detection hotspot

- This collection of signals was detected by our software half an hour either side of the primary swarm and results in the occurrence hotspot between 2 and 3pm on the 21st April 2015 recorded on the central accelerometer. In addition to DVAV signals, bees buzzing can also be heard as well a pipe perhaps originating from the queen.

S1 Video = High-definition video of a honeybee DVAV signal being performed in our observation hive.

- This is a short sample of video footage showing a DVAV signal being performed by an individual onto another bee. This film was shot in 1080p FHD at 50fps. The way the legs are used to grasp the recipient is described in the main manuscript.

S2 Video = High-definition video of a honeybee performing a shaking run in our observation hive.

- This is a short sample of video footage shows a shaking run being performed by an individual. The individual is tracked as she moves around the comb repeatedly performing DVAV signals until she is stopped by two nest mates. This film was shot in 1080p FHD at 50fps. Note that some DVAV signals are delivered directly onto the honeycomb.

S3 video = DVAV signal performed on the left accelerometer

- A video excerpt of a honeybee delivering a DVAV signal directly onto the accelerometer. The audible accelerometer data is also provided. A window containing a 2x zoom of the DVAV signal is interpolated and superimposed onto the video. The synchronised image of the acceleration of each individual abdominal knock demonstrating the typical Π-shape is also shown alongside the synchronised 2D-FT image showing 18Hz broadband peaks in spectral repetition. Note that the signaller actually holds a recipient bee even though the vibration is mostly delivered to the honeycomb by direct abdominal collisions.

S4 video = DVAV signal performed on the frame without protruding accelerometers

- A video excerpt of a honeybee delivering a DVAV signal onto the side of the frame without protruding accelerometers. The audible accelerometer data is also provided. A window containing a 2x zoom of the DVAV signal is interpolated and superimposed onto the video. The synchronised image of the acceleration of each individual abdominal knock demonstrating the typical C-shape is also shown alongside the synchronised 2D-FT image showing 22Hz broadband peaks in spectral repetition. Two DVAV signals occur, one is audible and the one that follows is not. Note that the DVAV signal is delivered onto brood cells partially becoming uncapped by emerging bees.

S5 video = Collection of DVAV signals with full vibrational quantitation

- Provided in this video is the analysis of the 27 high-SNR pulsed vibrations that were used in the initial vibrational quantitation of DVAV signals. The time course of the acceleration of the digital signal can be found in subplot (a), the image of the time course chopped into its individual knocks can be found in subplot (b), mean acceleration of a typical knock is provided provided in subplot (c) and the 2D-FT analysis can be found in subplot (d) throughout the video. For each signal, the audible trace from the raw accelerometer data is also provided. It can be seen that each elicits the characteristic Π-shape in the knocks and vertical broad bands at a spectral repetition corresponding to that of the number of knocks in subplot (a). The abdominal knocks can be seen to have either sharp positive or negative acceleration depending on the side of the frame the DVAV signal was performed.

S6 video = Inaudible DVAV signal with 2D-FT analysis

- A video excerpt of a honeybee delivering an inaudible DVAV signal directly onto the accelerometer. The accelerometer data is also provided. The synchronised image 2D-FT showing the broadband peaks in spectral relation can be observed too. This exhibits two expected vertical bands in spite of the low SNR preventing us from hearing the knocks. A window containing a 2x zoom of the DVAV signal is superimposed onto into the video.

S7 video = Video with Grid.

- This is a short sample of video footage with a grid superimposed over the top. The videos used in the analysis of spatial distribution had an identical grid superimposed. Whilst watching the videos, the coordinates of the abdomen of each individual performing a DVAV signal was recorded. All videos were in 1080p (full HD) definition at 50 frames per second. There is no audio in the video.

S8 video = Bee working in a cell.

- This is a short sample of video footage with the synchronised accelerometer data provided as a soundtrack. In the video, an individual honeybee is monitored as it works within a cell of the honeycomb. whilst we cannot see inside the cell, the individual's body movements appear to correlate with the high-amplitude clicks that can be heard on the accelerometer audio and presented in Fig S26. The video was recorded in 1080p (full HD) definition at 50 frames per second.

Video verification of DVAV signals detected within the accelerometer data associated with video footage from our observation hive.

Video Names	Date/Time of Video	Dur. (min)	Number of DVAVs	Detection Number	Correct Detections	% Genuine	Notes
**DSC0007 00006.MTS**	11:44am 8th July 2016	10	205	13	10	77	All over frame. No capped brood. **Hot and sunny day.**
**DSC0002 00007.MTS**	14:00pm 21st July 2016	10	0	0	0	100	0 occurred. 0 were falsely detected. Capped brood. Waggle Dances. **Hot and sunny day.**
**DSC0011**	12:05pm 29th July 2016	20	114	3	3	100	Fully capped brood and dense capped honey. 0 occurred within around 7cm of the accelerometer. 10 in range of the accelerometer, 4 of which were on top of other bees. Audible DVAV on 00010.MTS @ 34s. **Hot and Sunny day.**
**DSC0016 00017.MTS**	13:58pm 19h Aug 2016	20	19	1	1	100	2 in range of Acc. Capped Brood. Waggle Dances. On 00017.MTS, between 30 and 90s a bee DVAV signals until she receives a strange behaviour at the top of the frame and then does no more DVAVs. **Rainy day.**
**DSC0018 00019.MTS**	14:31pm 22nd Aug 2016	20	0	0	0	100	0 detections, 0 seen visually. **Rainy day.**
**DSC0021 00005.MTS**	16:00pm 30th Aug 2016	20	0	1	0	-	0 occurred. 1 was falsely detected. **Hot and sunny day.**
**DSC0024 00007.MTS**	11:20am 5th Sep 2016	20	95	1	1	100	Honeycomb almost empty. Some capped brood. Waggle dances occurring on both sides. No DVAVs occurred within the vicinity of the accelerometers other than 9:28 acc. 1. **Hot and sunny day.**
**DSC0005 00011.MTS**	11:00am 7th Sep 2016	20	30	0	0	100	Honeycomb almost empty. Some capped brood. All DVAVs transitioned into Waggle dances occurring on both sides. No DVAVs occurred within the vicinity of the accelerometers. **Warm and overcast day.**
**DSC0010 00024.MTS**	13:01pm 15th Sep 2016	20	20	0	0	100	Empty comb. 1 DVAV on the left accelerometer but it did not make contact with the comb. **Light rain and overcast.**
**DSC0012 00026.MTS**	10:00am 16th Sep 2016	20	11	0	0	100	Empty comb. 0 in range of accelerometer. **Heavy Rain and Fog.**
**DSC0013 00008.MTS**	15:10pm 28th Nov 2016	20	21	0	0	100	Very densely packed honey on comb. No waggle dances due to winter. 0 near the accelerometer. **Sunny day around 8°C.**
**DSC0015 00032.MTS**	16:21pm 19th April 2017	20	37	11	10	91	Honeycomb empty and very few bees on the frame. One side is completely void of bees and any contents within the frame. **Warm sunny day.**
**DSC_0013 00036.MTS**	14:00pm 9th Aug 2017	10	0	0	0	100	The frame was completely covered, on both sides, with capped brood. No DVAV signals occurred in this video. **Rainy day.**

Supplementary S1 Table. Outcome of DVAV signals that were detected automatically by our software compared to those visually observed upon the frame for 26 videos of 10 to 20minutes in length totalling a full time duration of 7.6 hours.

As seen above in Table 1, which displays the rate of successful detections of DVAV signals occurring within the accelerometer data associated with video footage from our observation hive, false detections are rare for the detection thresholds used to scan all datasets. For videos such as DSC0007, if the severity of the threshold values were reduced, the number of correct detections increases but so does the number of false detections also. This confirms that the detection threshold limit are at an optimum level for DVAV detection.

DVAV signals delivered upon opposite sides of the frame

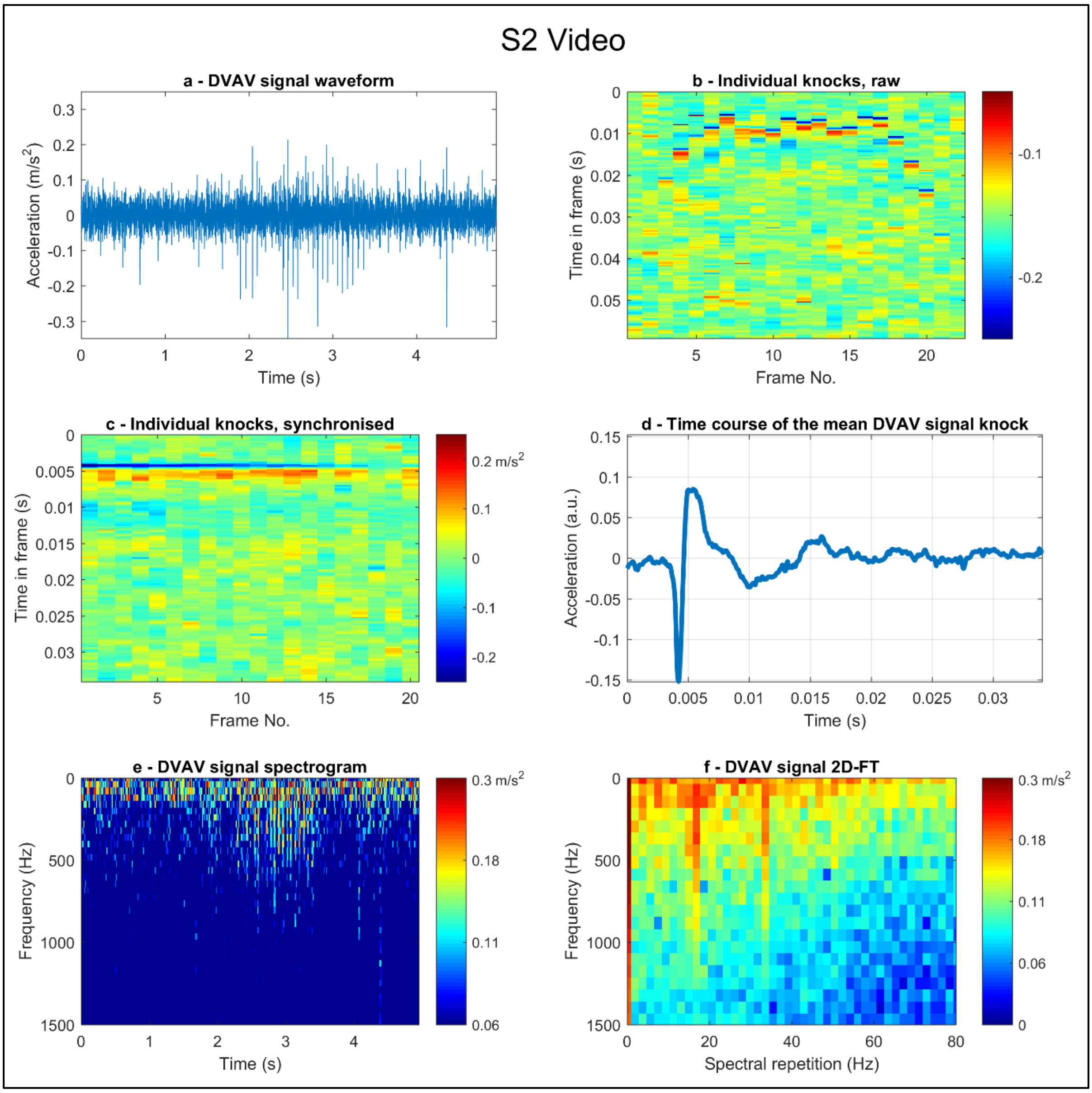

Fig S1. Vibrational properties of the DVAV signal delivered onto the side of the frame with the accelerometers as seen in Video S2. (a) Time course of honeycomb acceleration waveform during the delivery of a DVAV signal; (b) The same waveform, shown in successive frames adjusted to the time gap residing between two individual abdominal-honeycomb collisions; (c) The DVAV signal knocks, aligned to the first one; (d) The time course of the mean of the DVAV signal knocks; (e) The spectrogram of the complex DVAV waveform in Fig S1a; (f) The 2D-FT image of the complex DVAV waveform in Fig S1a. The colour bar displays the linear scale amplitude in m/s2.

In Fig S1a we show the complex waveform of the DVAV signal in Video S2 that occurred directly on top of the left accelerometer. The signal can be observed to last around one second and contains seventeen individual knocks (Fig S1b) that form the characteristic Π-shape when segregated into equal length frames around each of the individual “knocks”. Upon averaging of the individual abdominal knocks, it can be seen that the sharp bursts of acceleration associated with DVAV signals all have a negative polarity direction as opposed to a positive polarity as in Fig S2 for the DVAV signal that

occurred on the other side of the frame. Similar oscillations can be observed as in Fig 1 as the honeycomb relaxes but this is much weaker due to the greater distance between the sensor and the signaller. The 2D-FT also shows that this signal has a frequency of 16.9 Hz with upper harmonics at 34 and 52 Hz.

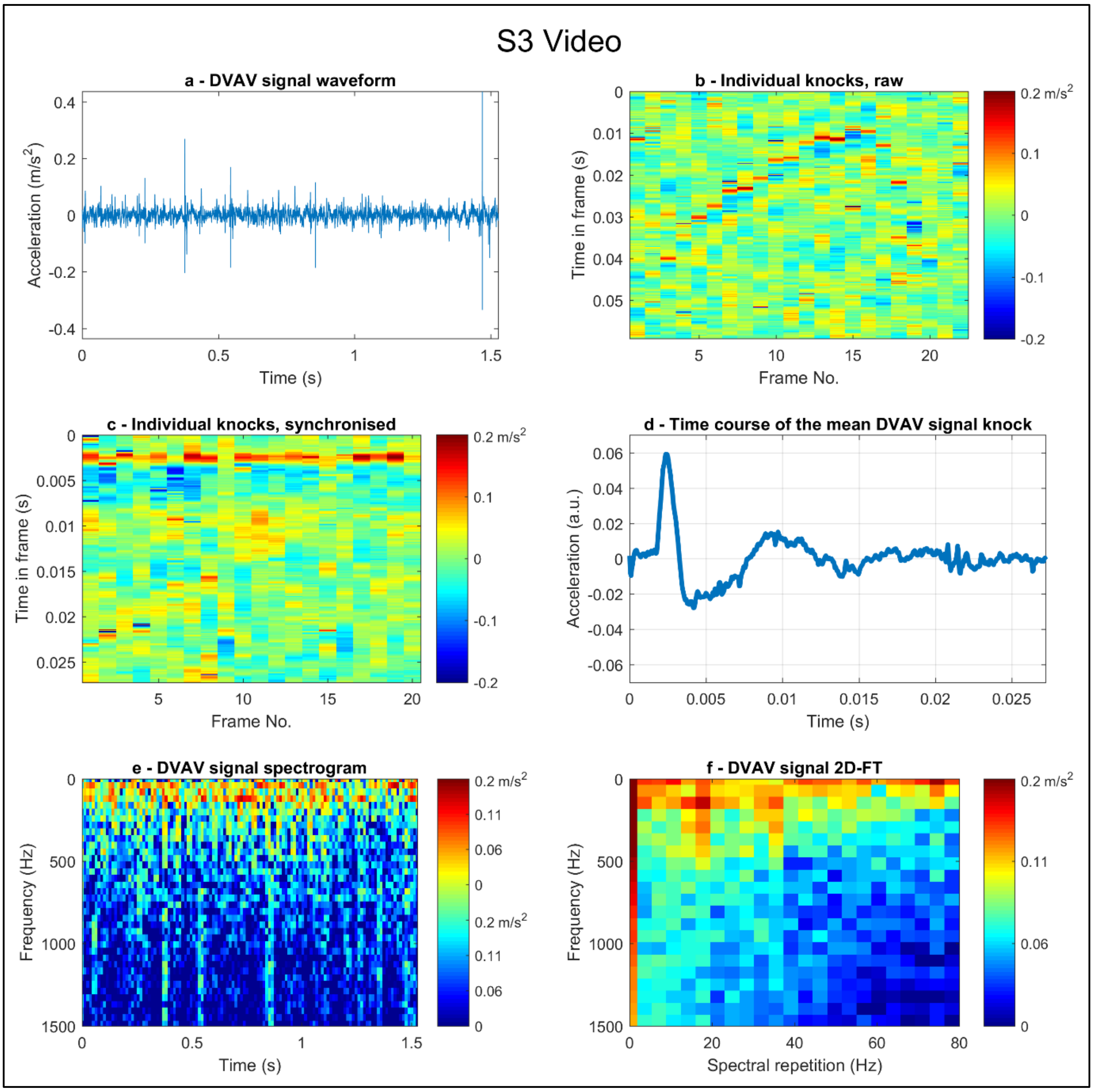

Fig S2. Vibrational properties of the DVAV signal delivered onto the side of the frame to the reverse of the accelerometer as seen in Video S3. (a) Time course of honeycomb acceleration waveform during the delivery of a DVAV signal; (b) The same waveform, shown in successive frames adjusted to the time gap residing between two individual abdominal-honeycomb collisions; (c) The DVAV signal knocks, aligned to the first one; (d) The time course of the mean of the DVAV signal knocks; (e) The spectrogram of the complex DVAV waveform in Fig S1a; (f) The 2D-FT image of the complex DVAV waveform in Fig S2a. The colour bar displays the linear scale amplitude in m/s2.

In Fig S2a, the complex waveform of the DVAV signal in Video S3 that occurred on the opposite side of the frame to the visible accelerometers and cables can be seen. As in Fig S1, the signal can be observed to last around one second and contains twenty-two individual knocks (Fig S2b) that end abruptly as the signaller falls from the honeycomb (Video S3). Upon averaging of the individual abdominal knocks, it can be seen that the sharp bursts of acceleration associated with DVAV signals occurs in a positive direction as opposed to a negative direction as in Fig S1 for the DVAV signal that occurred on the other side of the frame. The signal in Fig S2 has a much lower amplitude than that of the DVAV signal that occurred directly on top of the accelerometer on the other side due to the accelerometer being placed on that face of the honeycomb and not wedged directly in the centre. Similar oscillations can be observed as in Fig S1 as the honeycomb relaxes but this is much weaker due to the greater distance between the sensor and the signaller. The 2D-FT also shows that this signal has a frequency of 18Hz with upper harmonics at 34 and 52 Hz. This, in combination with Fig S1, shows that DVAV signals can be detected on both sides of the frame and that the polarity of the acceleration reveals the side of the frame where the signaller resides.

Vibrational quantitation of honeybee clicks

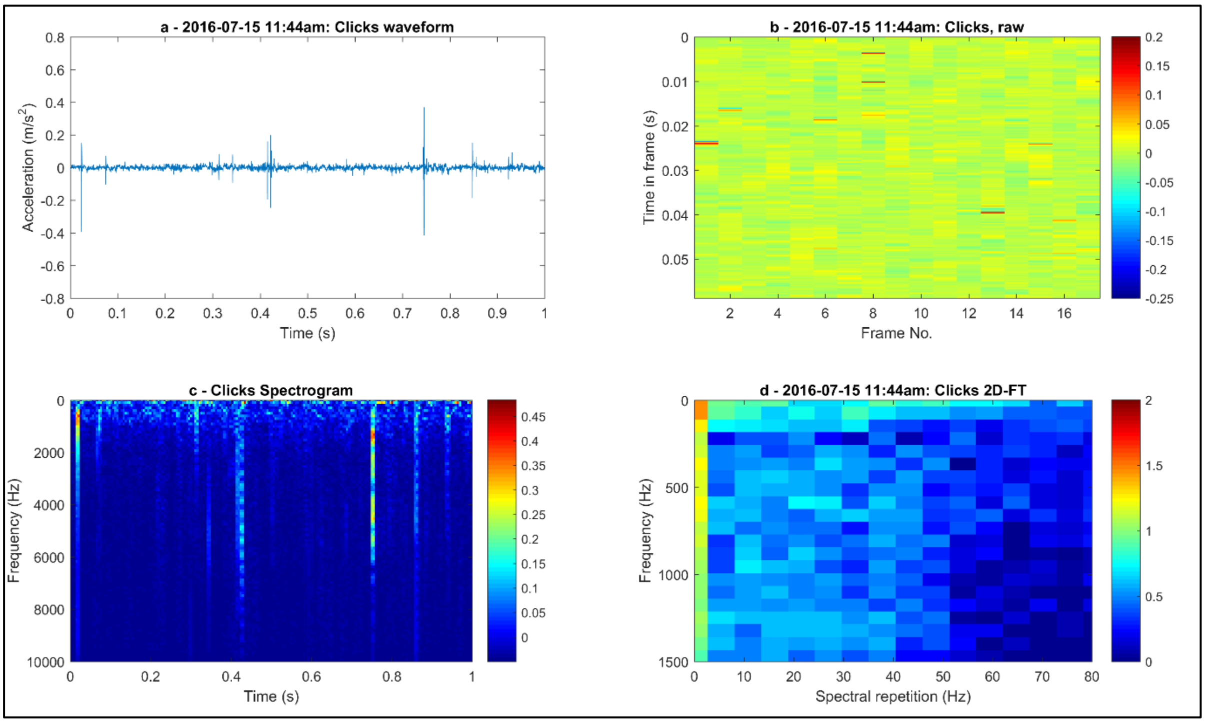

Fig S3. Vibrational properties of a series of high-amplitude clicks extracted from data associated with S6 Video. (a) Time course of honeycomb acceleration waveform during the detection of the signal; (b) The same waveform, shown in successive frames adjusted to 55ms; (c) The spectrogram of the complex the accelerometer trace in Fig S3a; (d) The 2D-FT image of the waveform in Fig S1a. The colour bar displays the linear scale amplitude in m/s2.

Video evidence suggests that these particular clicks are the result of a honeybee working at the bottom of an empty cell and have been extracted from the raw accelerometer data associated with S6 Video. It can be seen in Fig S3a that the individual clicks produce high amplitude spikes of similar magnitude to the individual knocks of the DVAV signal. However, as further highlighted in Fig S3b, they are sporadically distributed along the time axis and do not exhibit the characteristic Π–shape associated with DVAV signals. The spectrogram in Fig S3c shows that, like a single knock of a DVAV signal, the individual clicks exhibit a broad band spectrum of frequencies. However, unlike the DVAV signal where the majority of relevant information exists below 2500 Hz (Fig S4), the broadband spectra of the clicks can extend up to 10 kHz. In addition, due to the irregularity in the distribution of the clicks within the time domain, the 2D-FT analysis (Fig S3d) shows no vertical bands at a specific spectral repetition even though in this particular example 13 clicks were recorded over 1 second (the duration of a typical DVAV signal).

Spectral analysis of individual DVAV signal knocks

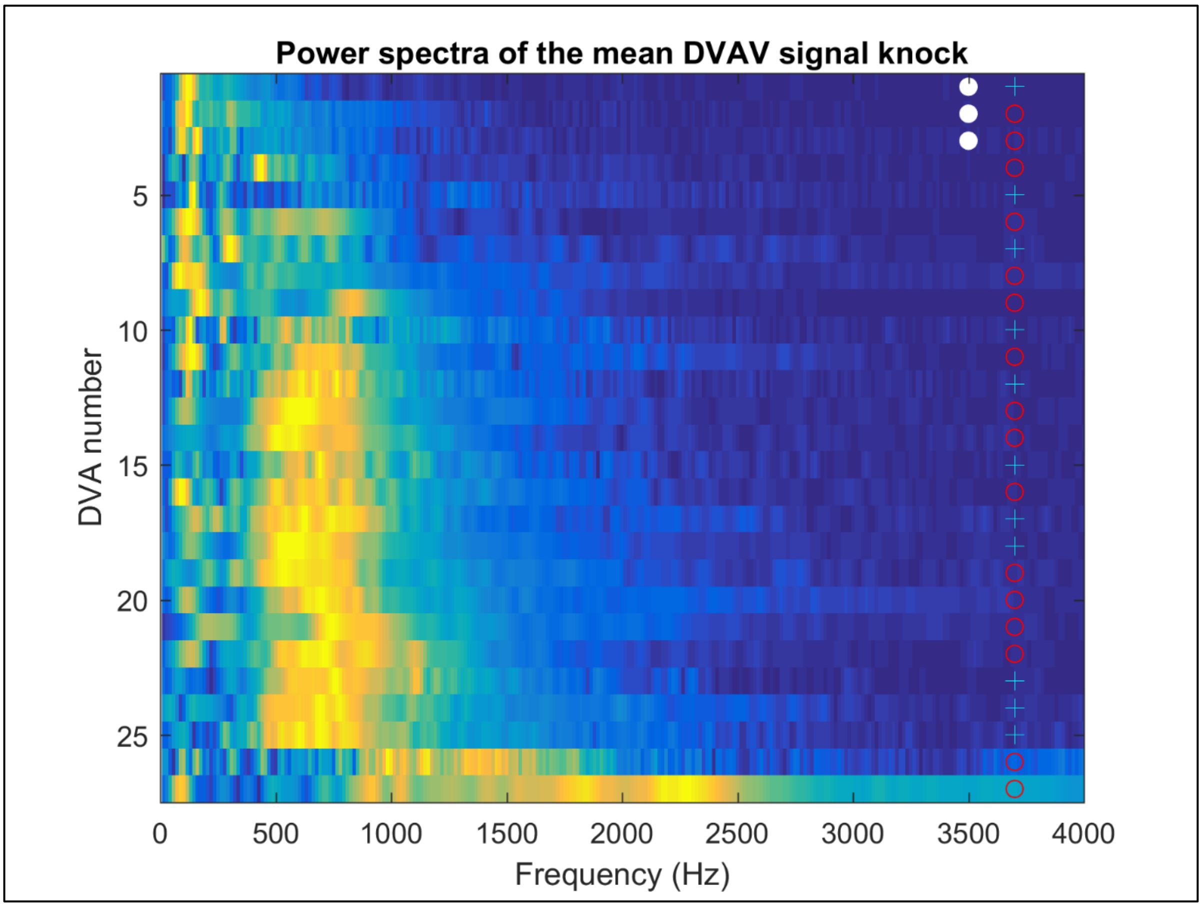

Fig S4. The power spectra of each averaged knock of the twenty-seven high SNR pulses used in the initial quantitation of DVAV signals (see S4 Video). These pulses have been ordered by their increasing similarity to the population mean. Pixel intensity denotes the acceleration amplitude of each frequency. White dots show DVAV signals that were extracted from the Clifton Observation hive. The remaining pulses are from before the primary swarm in the French 2015 dataset. The cyan + symbol marks a pulse with knocks that are of positive polarity and the red O symbol marks a DVAV signal that has knocks that are of negative polarity.

In Fig S4 we show the power spectra of each averaged knock of the twenty-seven high SNR pulses used in the initial quantitation of DVAV signals. Principal component analysis was used to order the pulses by decreasing distance to the population mean. It can be seen that the majority of pulses have a sharp peak at around 60Hz, characteristic of a log-lasting slow oscillation, and a secondary broadband peak, that is characteristic of a short lived ultra-fast oscillation, between 500 and 1000 Hz. There appears to be little relevant information above 1500 Hz for the majority of DVAV signals. The polarity of the pulses, caused by the side of the frame upon which the signal was delivered, appears to have no effect on the spectra of the pulse's mean knock. Fig S4 also shows that the DVAV signals that come from other hives to that of the majority are outliers to the rest of the population. This further supports the concept that the current status of the honeycomb influences the frequency response of its relaxation caused by the individual abdominal–honeycomb collisions that make up the DVAV signal.

DVAV signal occurrences – Clifton Observation hive

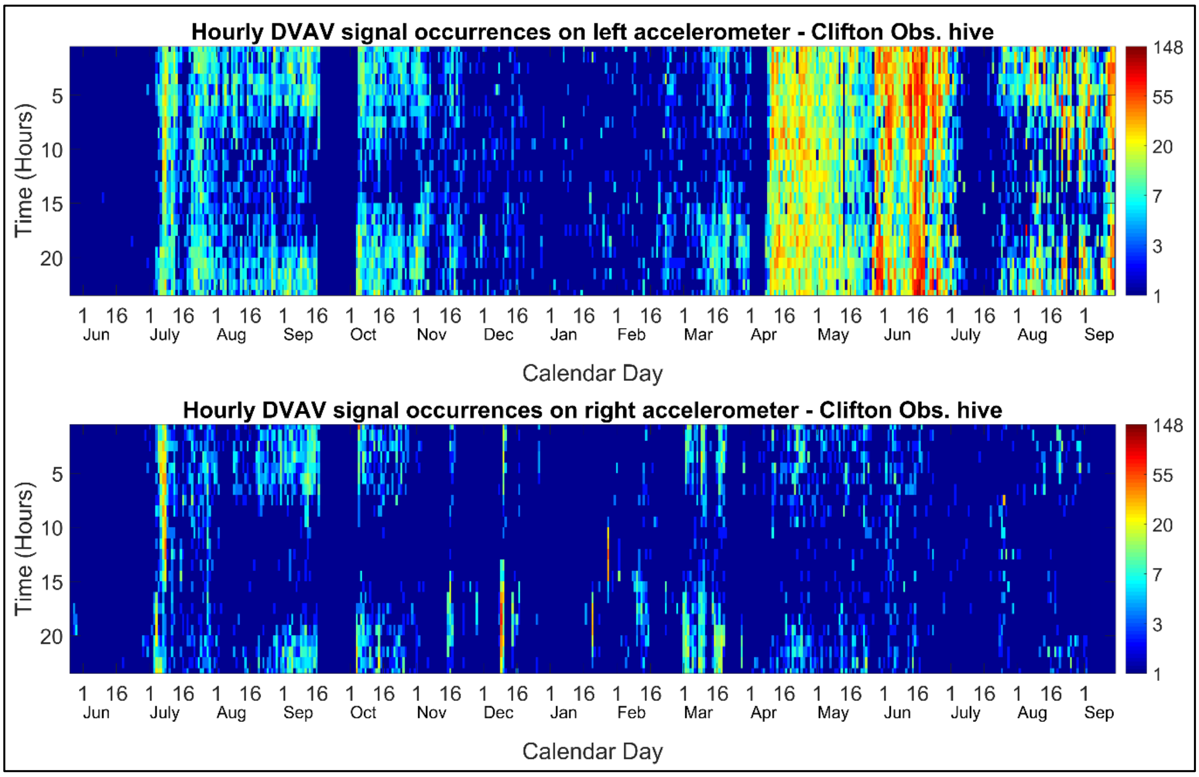

Fig S5. DVAV signal hourly occurrences. Left (top) and right (bottom) accelerometer logs of the Clifton observation colony (2016/17 season). The colour codes the number of hourly occurrences from dark blue (1) to dark brown (148 signals) on a logarithmic scale.

The dataset for the Clifton Observation hive was situated on the Clifton Campus of Nottingham Trent University, Nottingham, UK. This colony experienced many natural and human induced changes throughout its period under observation. The colony was introduced to its new observation hive on the 24th May 2016. It was placed into the box via a purpose cut hole on the left side. From there the colony developed across the box and began work on the observation frame on the 5th July 2016. On 16th September 2016, the colony was removed from campus for 3 weeks so that they could be brought back and situated in another building, resulting in the 3-week interruption to the measurement seen in September. The recordings then continued across the winter until 28th March 2017 when the hive was brought back to the lab for cleaning and replacement of the honeycomb with fresh foundation wax, causing a weeklong interruption to the signal. The hive was arranged so that the observation frame was placed at the centre of the colony, which causes the increase in the number of detections during this period. The right accelerometer was accidentally placed so that its axis faced parallel to the honeycomb, instead of perpendicular and thus the detection of the DVAV signal is much less frequent on the right channel compared with the left. This experimental mistake demonstrates the advantage of setting up the uniaxial accelerometer’s measurement axis normal to the plane of the honeycomb. Additionally, the left accelerometer was much more exposed and surrounded by empty cells in contrast to the right accelerometer that was buried deep surrounded by capped honey stores.

This frame was at the periphery of the colony throughout most of the recordings and makes for an interesting comparison to the central frames of the 2015 and 2017 French datasets. The brood cycle (which is usually only takes place on the four to five frames most central to the colony) is not regularly seen on this dataset and this frame only experienced one in August 2016 and one in July and August 2017. However, similar trends such as the signal reduction towards winter with the increase in May and drop off in July that we see on the French 2015 dataset can be observed in this dataset.

The queen of this colony was old and became an inefficient egg-layer. On 16th June 2017, the old queen was found in the observation unit, trying to escape the colony. On inspection, queen cells were found within the frames of the colony. A few days later, the original queen was found dead outside the hive and a new queen was seen within. This dual-queen transitional period could be the cause of the drop in DVAV signal occurrences that detected in July 2017.

On the 9th August 2017, a video was recorded of the observation frame for 10 minutes in which no DVAV signal occurred on a frame that included honey, empty cells and capped brood. The associated accelerometer track exhibits numerous high-amplitude clicks. However, there was no false detections recorded by our software in this period giving further confidence in the success of the detection algorithm.

DVAV signal occurrences – French 2017 hive

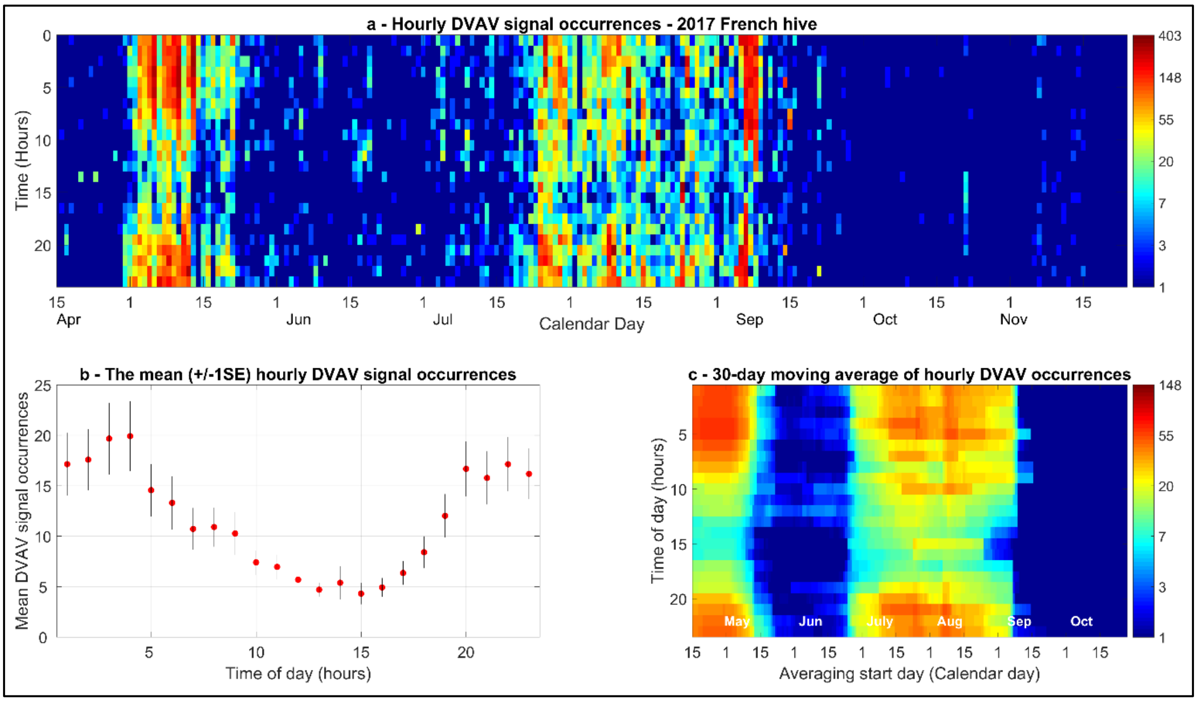

Fig S6. The hourly occurrence statistics for the 2017 French hive dataset showing: (a, top) the histogram of the hourly occurrences of the DVAV signal for each day of recording, (b, bottom left) the mean number of DVAV signal detections for each hour of the day throughout the dataset, and (c, bottom right) the number of DVAV signals that occurred for each hour of the day averaged over 30-days and shifted along the dataset in increments of one day.

Presented in Fig S6 is the hourly occurrence statistics for the French 2017 hive dataset, which spans the entire 2017 active season until the failure of the colony found on the 28th November 2017. This hive was monitored using one accelerometer placed directly into the centre of the honeycomb pertaining to the hive's most central frame. From when the recording was launched on the 15th April 2017, there were very few DVAV signals detected until the 29th April when we see a large increase in the number of DVAV signals that is also apparent on the French 2015 dataset. This was data ascertained from a hive where the colony did not swarm and as a result, there is no increase in DVAV signals detected in early April as seen for the 2015 French dataset in Fig 4, which supports the DVAV signal's association with pre and post swarming practices. As with all other datasets (Figs 4 and S5), there is heightened signal detection in July, yet there is no 21-day brood cycle apparent within this dataset. However, there is a regular 12-day peak in DVAV signal detection suggesting an asynchrony of laying and emergence between each side of the frame or could indicate the presence of drone-laying workers. The number of DVAV signals averaged for each hour of the day across the entire 2017 French dataset (Fig S6b) shows the same 10am-4pm minima and 10pm – 4am maxima as seen across all other datasets. This "lunchtime lull" is seen to hold steady across the dataset until the collapse of the colony (Fig S6c).

Comparison between the daily trends of whooping signal occurrences, DVAV signal occurrences and the brood cycle as displayed by the modal night time amplitude

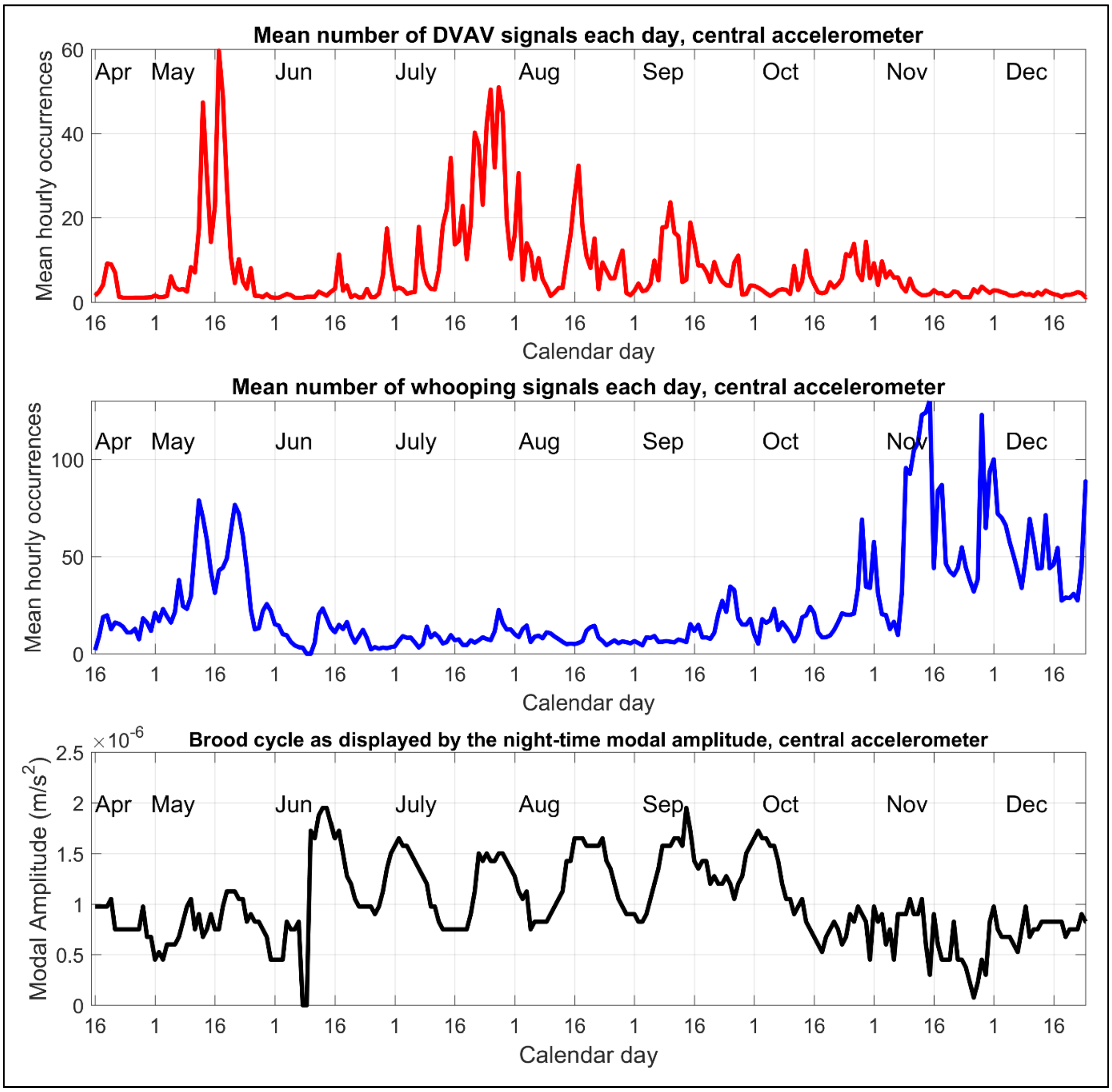

Fig S7. Comparison of the daily trend in the mean number of hourly DVAV signals (red), whooping signals (blue), and the night-time modal amplitude (black), for the central accelerometer of the 2015 French dataset.

The comparison between the daily trends of DVAV signals, whooping signals and the brood cycle data published in Ramsey et al. (2017) for the French 2015 hive dataset is shown in Fig S7 for the central accelerometer. There is a significant positive correlation between the modal midnight amplitude of (Bencsik, et al., 2015) the entire dataset and the daily mean number of DVAV signals recorded by our software on both the central (Fig S7) (R_s = *0.2475, p <0.001*) and peripheral (Fig S8) (R_s = *0.3505, p <0.001*) accelerometers, with peaks in both every 21–24 days. When the modal amplitude peaks (after the hatching of brood) the number of DVAV signals increases. This trend, however starts with the second brood cycle; not seen after the first hatching on the 7th June. There is a final DVAV signal

occurrence peak 21 days after the last hatching of brood on the 4th October, a feature seen on both accelerometers.

Interestingly, there is no correlation between the hourly number of DVAV and whooping signals (from Ramsey *et* al., 2017), recorded on the central (Fig S6) ($R_s = 0.0735, p = 0.2461$) or peripheral ($R_s = 0.256, p = 0.1002$) accelerometer (Fig S7). There are peaks in DVAV and whooping signals, moat evident in the peripheral accelerometer data, that are concordant, such as around the 17th May, however the peaks in DVAV signals always occur around 3 days before that of the whopping signals. There is also an increase in the occurrence of whooping signals in November and December that is not seen for the DVAV signals. The whooping signals also exhibit a peak in occurrence around the 11th June that is in line with the first brood cycle, a feature that is not evident within the occurrences of DVAV signals.

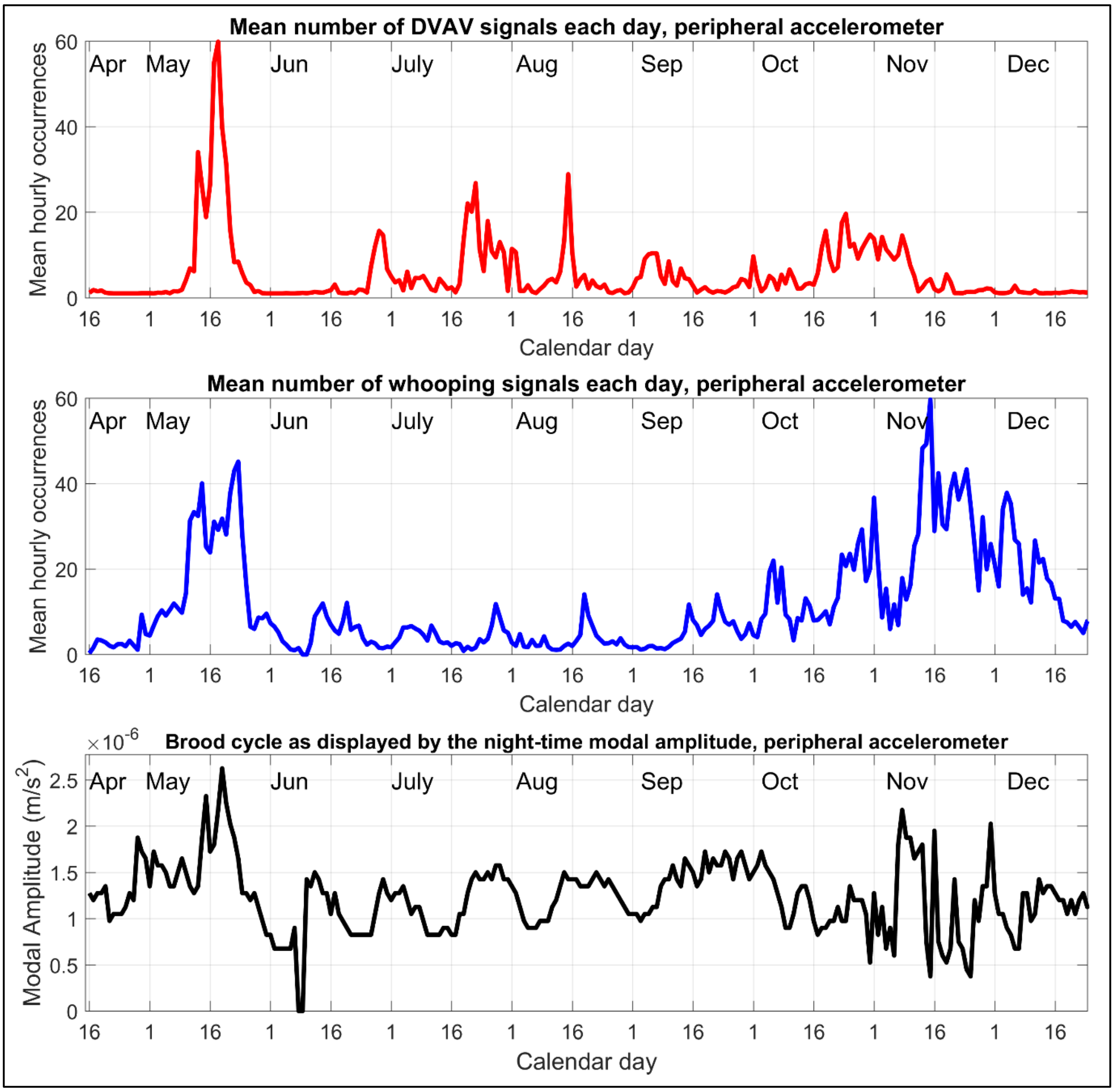

Fig S8. Comparison of the daily trend in the mean number of hourly DVAV signals (red), whooping signals (blue), and the night-time modal amplitude (black), for the peripheral accelerometer of the 2015 French hive dataset.

Hourly DVAV signal statistics – Clifton Observation hive

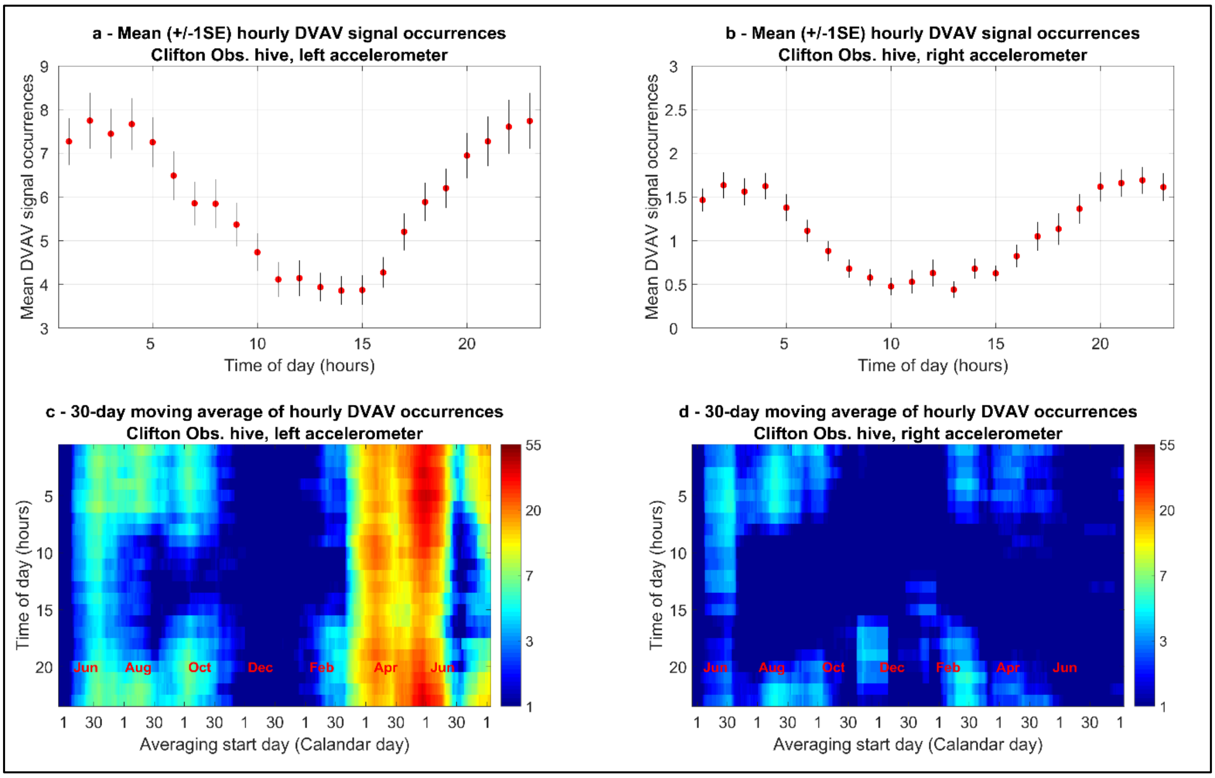

Fig S9. Average hourly occurrences within the Clifton Observation hive dataset. a) The mean hourly trend in DVAV signals detected by our software over 24 hours across the entire dataset, recorded on the left accelerometer and b) on the right accelerometer; c) The mean hourly trend calculated over 30 days and moved in increments of one day coming from the left accelerometer dataset and d) coming from the right accelerometer dataset. The vertical bars indicate +/- 1 standard error (SE).

The hourly statistics of the Clifton Observation hive (Fig S9) mirror that of the 2015 French dataset (Fig 5) and also that of the 2017 French dataset (Fig S6) with maxima in the evening and minima between the hours of 10am and 4pm (Fig7a), which holds stable across the entirety of the recording (Fig4b). Fig S9 further demonstrates the reduction in the number of detected DVAV signals on this (mostly) peripheral frame compared to the central frame of the other datasets. It suggests that the same trends in signal occurrence can be observed across the other frames of the hive and is not restricted to the one most central to the colony.

Hourly 2D-FT analysis

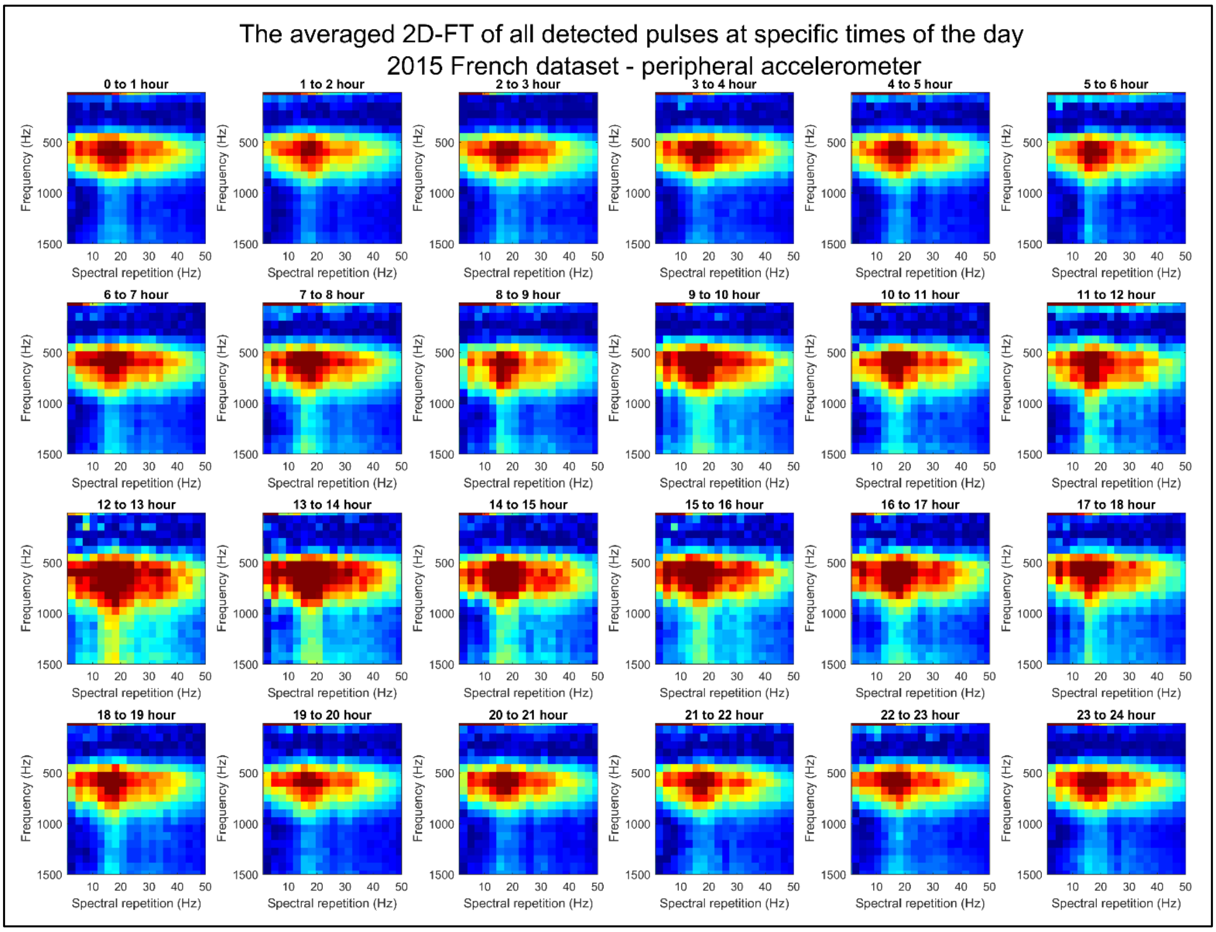

Fig S10. The mean 2D-FT of all DVAV signals detected on the peripheral accelerometer by our software for each hour of the day throughout the full 2015 French dataset. Pixel intensity from dark blue (low) to dark red (high) denotes the amplitude in arbitrary units.

In-line with that of the central data (Fig 6), the hourly 2D-FT average over the peripheral accelerometer data for the 2015 French hive (Fig S10) displays no effect of the time of day on the spectral repetition of the detected DVAV signals, with the frequency of the broadband spectrum centred predominantly around 18Hz. However, the amplitude of the broadband of frequencies between 500 and 1500Hz associated with the DVAV signal increases gradually from midnight and peaks between 12 and 2pm then gradually decreases again towards midnight. This trend is further evident for this accelerometer upon averaging the amplitude in the above 500Hz for a spectral repetition within 13 to 25 Hz band, as shown in Fig 7b.

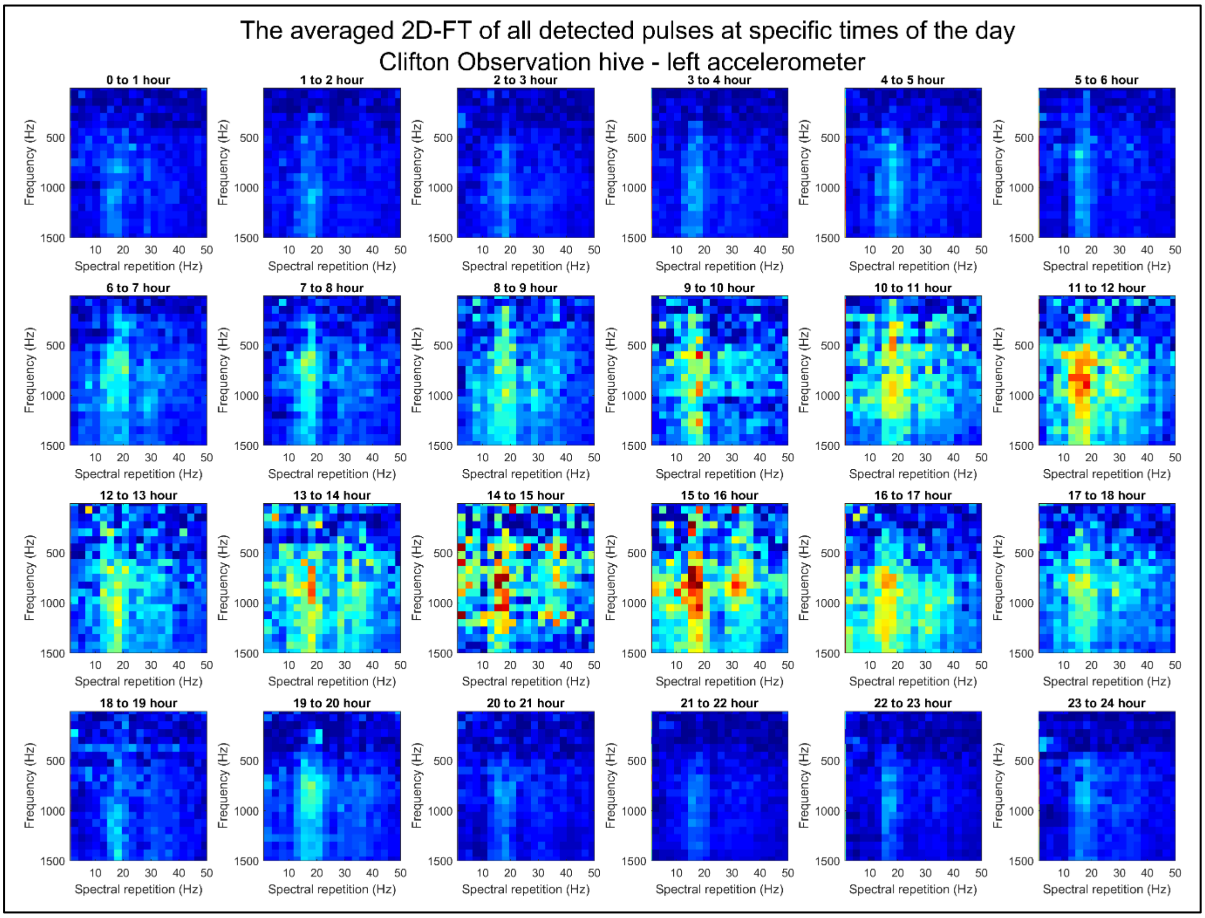

Fig S11. The mean 2D-FT of all DVAV signals detected on the left accelerometer by our software for each hour of the day throughout the full Clifton Observation hive dataset. Pixel intensity from dark blue (low) to dark red (high) denotes the amplitude in arbitrary units.

In-line with that of the central (Fig 6) and the peripheral data (Fig S10) for the 2015 French hive, the mean hourly 2D-FT of DVAV signals that occurred within the Clifton Observation hive dataset displays no effect of the time of day on the spectral repetition of those pulses detected by the left accelerometer (Fig S11) or the right accelerometer (Fig S12), with the repetition frequency of the broadband spectrum being predominantly centred around 18Hz. However, the amplitude of the broadband of frequencies between 500 and 1500Hz associated with the DVAV signal increases gradually from midnight and peaks between 2 and 4pm then gradually decreases again towards midnight, a trend that is inverse to that of the hourly occurrences data (Fig S9a). This trend is further evident for this accelerometer upon averaging the amplitude in the above 500Hz for a spectral repetition within 13 to 25 Hz band in S15 Fig. The 2DFT image becomes increasingly noisy around 2pm due to the reduction in the number of pulses detected for these hours across the entire dataset seen in Fig S4.

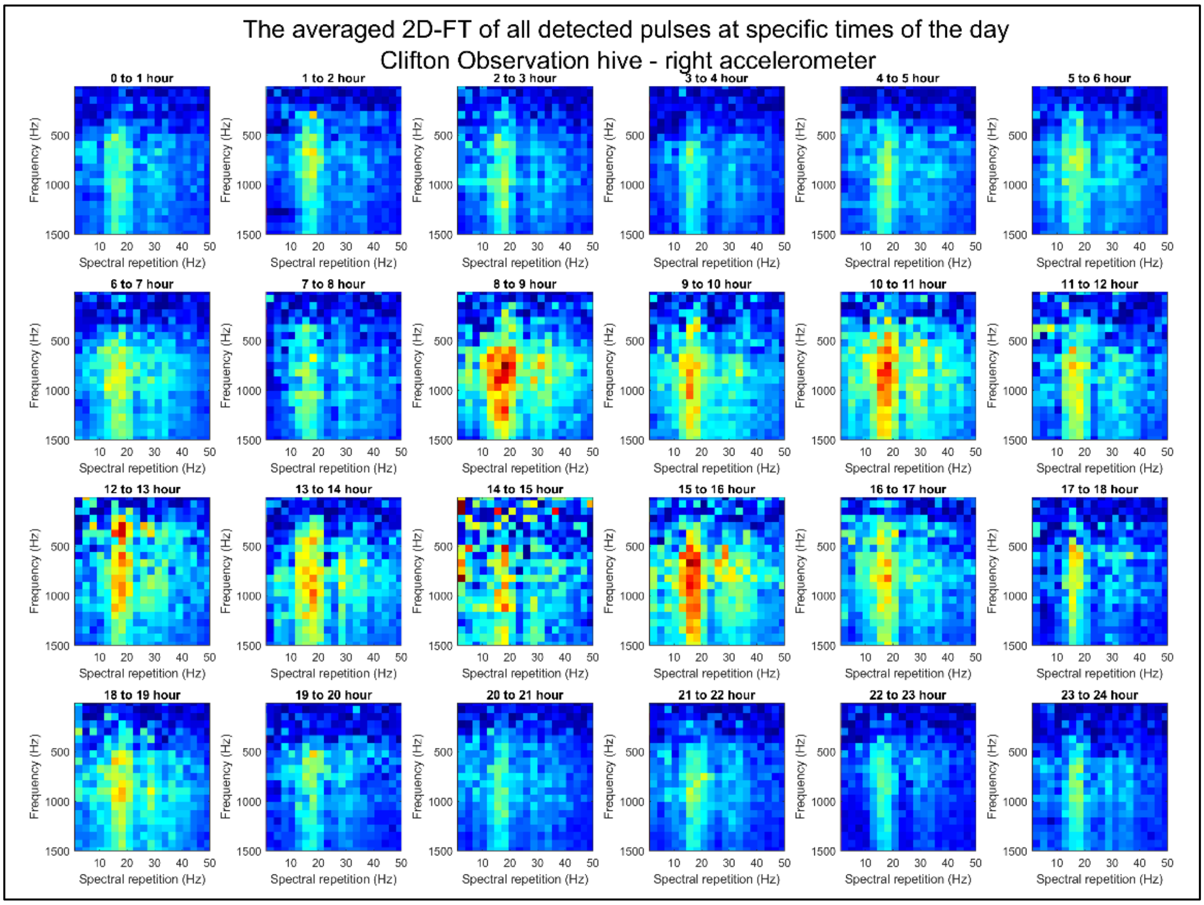

Fig S12. The mean 2DFT computed over all DVAV signals detected on the right accelerometer at each hour of the day across the entire Clifton Observation hive dataset. Pixel intensity from dark blue (low) to dark red (high) denotes the amplitude in arbitrary units.

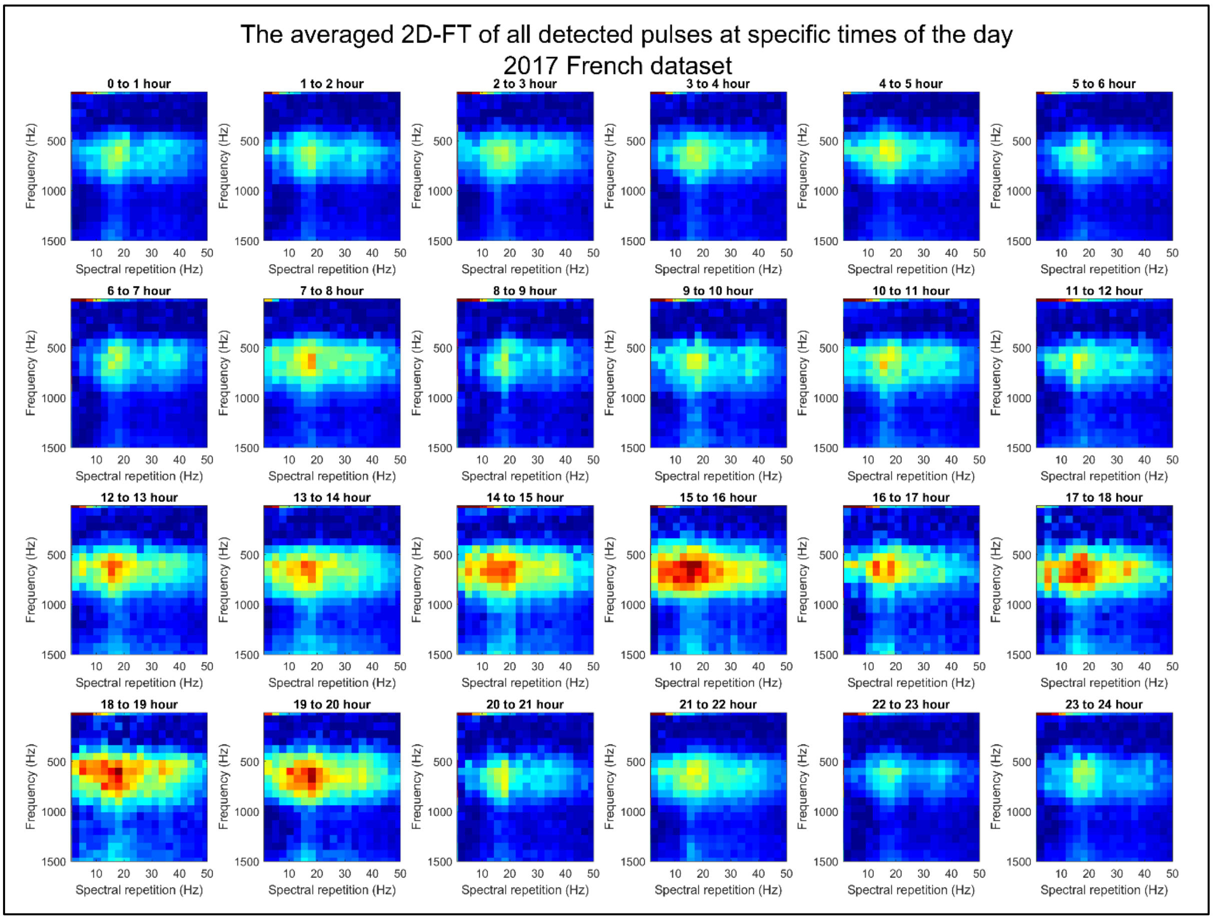

Fig S13. The mean 2D-FT of all DVAV signals detected on the centrally placed accelerometer by our software for each hour of the day throughout the full French 2017 hive dataset. Pixel intensity from dark blue (low) to dark red (high) denotes the amplitude in arbitrary units.

In-line with that of the central (Fig 6) and the peripheral data (Fig S10) for the 2015 French hive, the hourly 2D-FT average for the 2017 French hive data (Fig S13) displays no effect of the time of day on the spectral repetition of the detected DVAV signals, with the frequency of the broadband spectrum centred predominantly around 18Hz. However, the amplitude of the broadband of frequencies between 500 and 1500Hz associated with the DVAV signal increases gradually from midnight and peaks between at 4pm, remains high until 8pm then gradually decreases again towards midnight, a trend that is inverse to that of the hourly occurrences data (Fig S5a). This trend is further evident for this accelerometer upon averaging the band of frequencies above 500Hz for a spectral repetition of 14 to 25Hz in Fig S14.

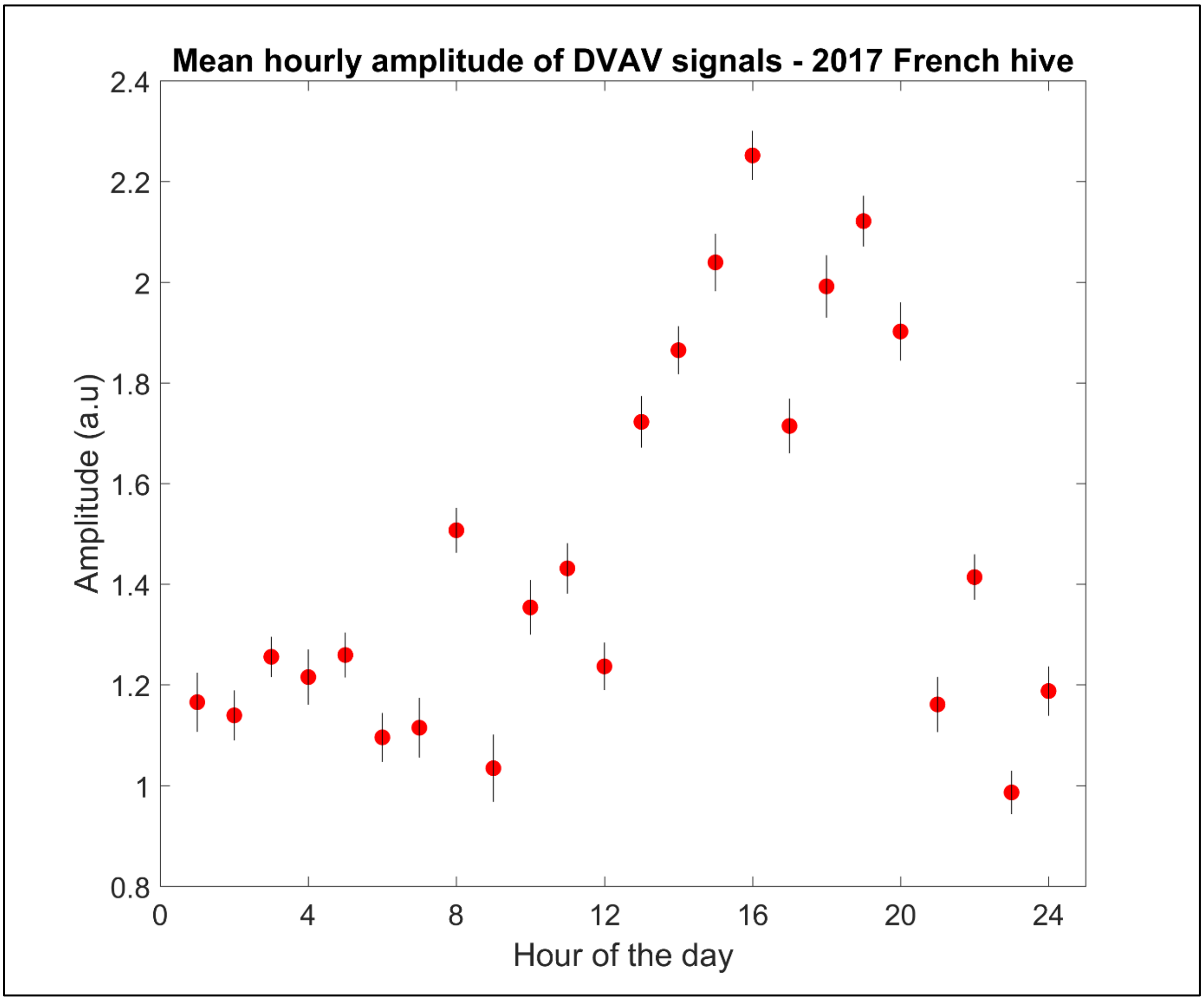

Fig S14. The mean (±1SE) hourly amplitude for the 14-25Hz horizontal spectral band of the DVAV signals detected within the French 2017 hive dataset

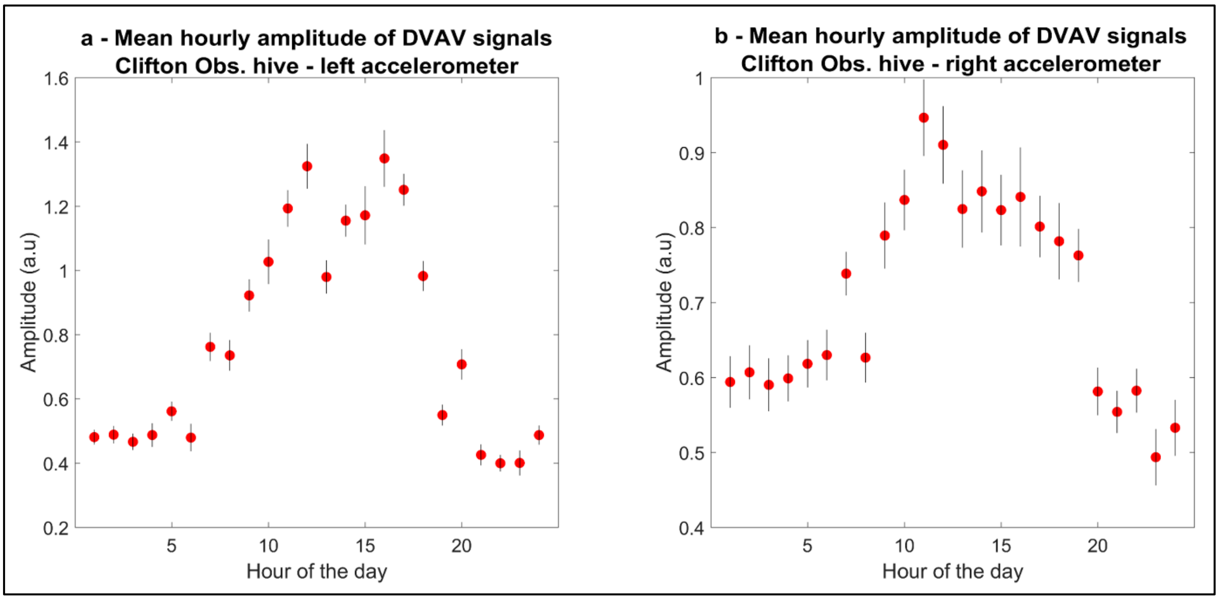

Fig S15. The mean (±1SE) hourly amplitude for the 14-25Hz horizontal spectral band of the DVAV signals detected within the Clifton Observation hive dataset for a) the left and for b) the right accelerometer.

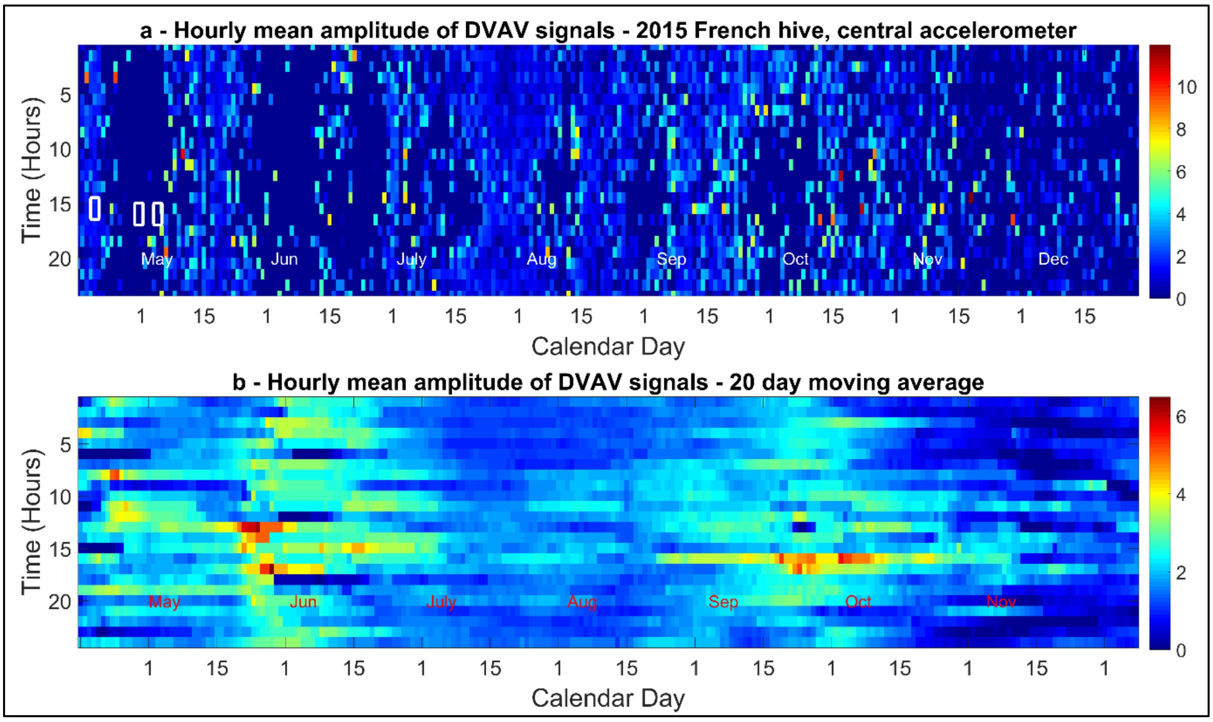

Fig S16. The mean amplitude of DVAV signals (a) calculated for each hour of recording; and then (b) averaged horizontally over a twenty-day long window for the detections by the central accelerometer of the French 2015 hive dataset.

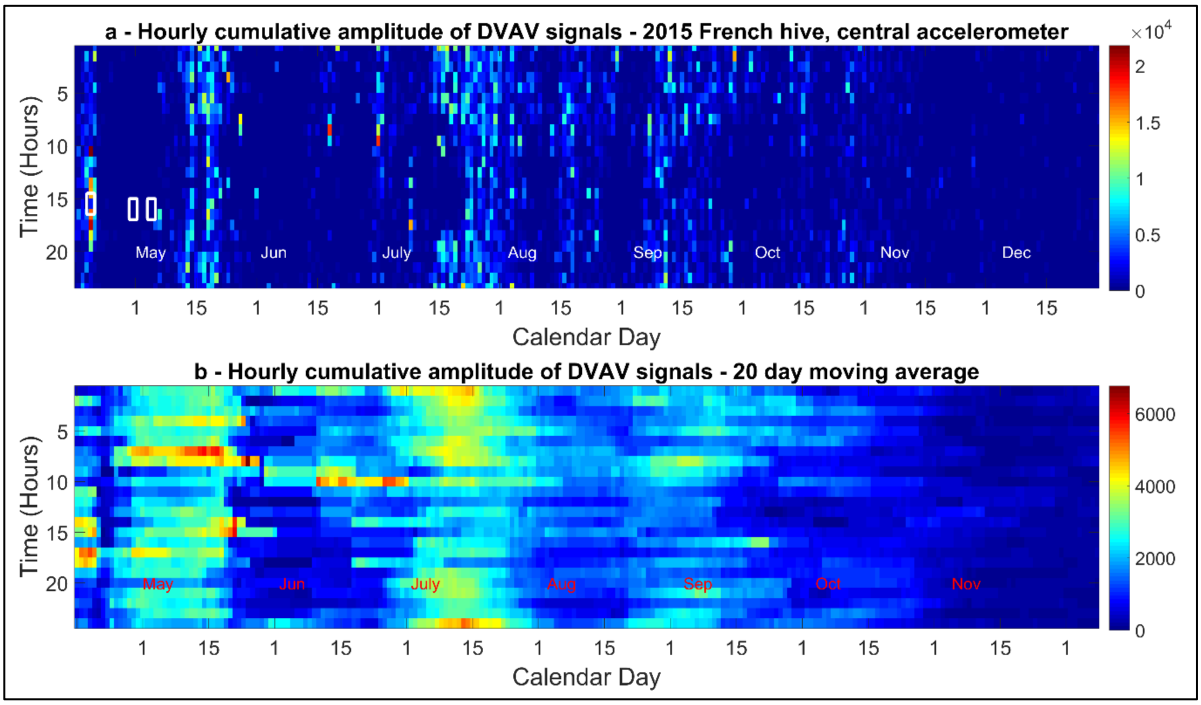

Fig S17. The amplitude of DVAV signals that are (a) summated over each hour of recording; and then (b) averaged over a twenty-day long window for the detections by the central accelerometer of the French 2015 hive dataset.

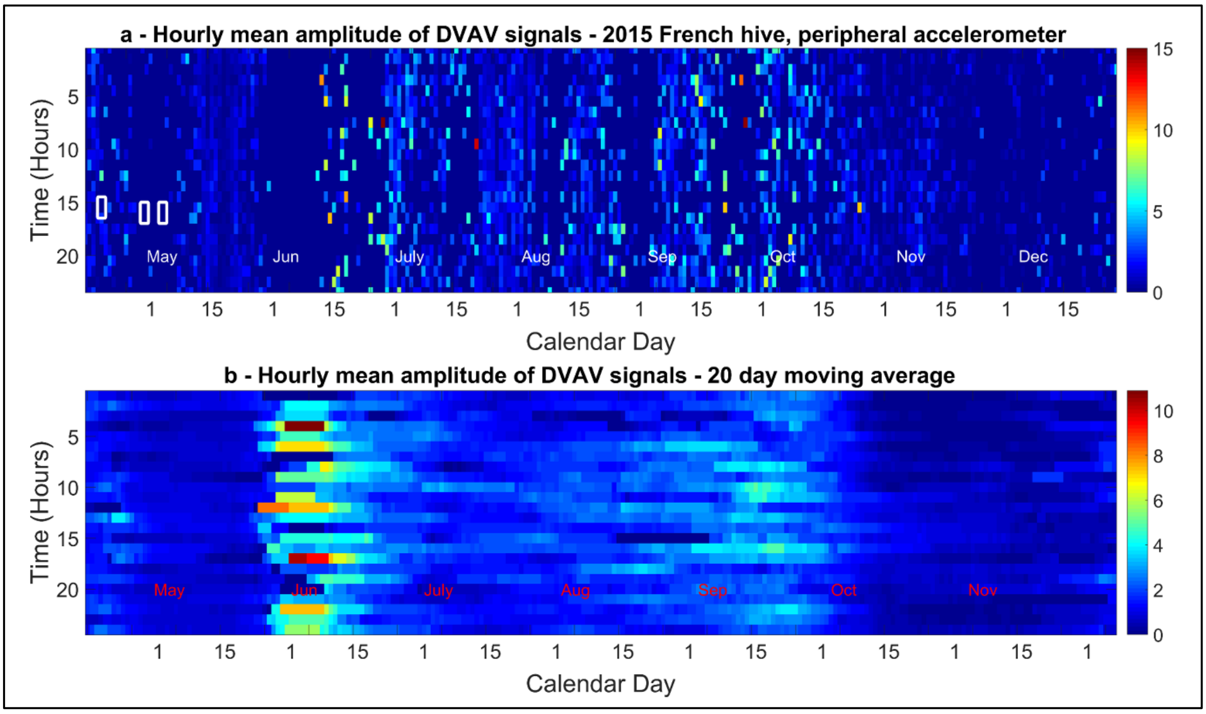

Fig S18. The mean amplitude of DVAV signals (a) calculated for each hour of recording; and then (b) averaged horizontally over a twenty-day long window for the detections by the peripheral accelerometer of the French 2015 hive dataset.

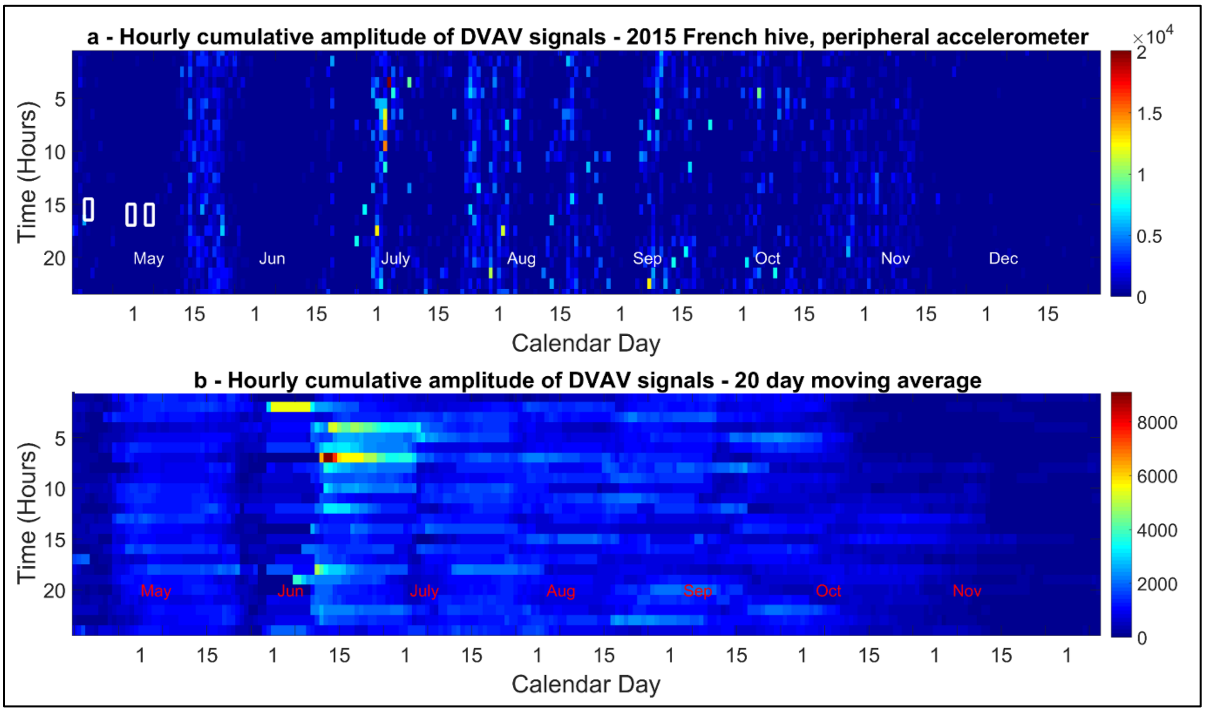

Fig S19. The cumulative amplitude of DVAV signals that are (a) summated over each hour of recording; and then (b) averaged over a twenty-day long window for the detections by the peripheral accelerometer of the French 2015 hive dataset.

Upon averaging the hourly amplitudes of DVAV signals horizontally across the entire 2015 season (Fig 6), the amplitude differences between those signals that were detected at night and those that were detected in the middle of the day is easily highlighted. However, as seen in Fig S16a and S17a, it is difficult to see this trend on a daily basis. It is only upon averaging the data across enough days (e.g. 20, Fig S16b and S17b), that this trend becomes more apparent. In other words, we have not detected enough DVAV signals throughout an average day to show confidently that this trend definitely occurs throughout each day of the recording.

Cumulating the amplitudes of the DVAV signals detected for each hour (Fig S18a and S19a), rather than averaging, allows to highlight instances of DVAV signals that are both numerous ***and*** strong. At 11am on the 20th April 2015, the highest sustained cumulative amplitude of our entire recording can be observed, and this is followed by the hours either side of the primary swarm, confirming that these sections of recording contain the highest temporal density of high SNR DVAV signals (>50 per hour). In the hour containing the primary swarm, there are no DVAV signals detected and thus the cumulative signal amplitude is low. When summating the hourly amplitudes (Fig S18b and S19b) of DVAV signals, the opposite trend can be observed compared to the computation of the mean (Fig S16b and S17b), due to more DVAV signals having been detected at night (around 3x), as compared to the middle of the day (Fig 4).

Daily 2D-FT analysis

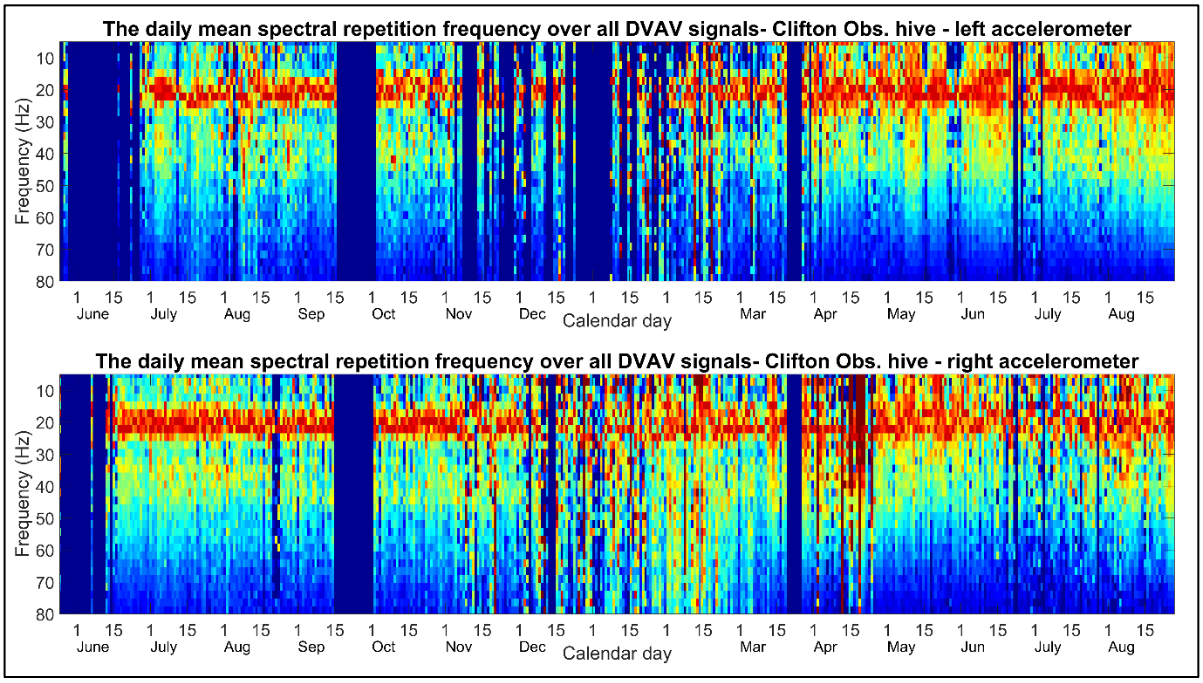

Fig S20. The daily mean spectral repetition of the DVAV signals detected within the Clifton Observation hive dataset for (a) the left accelerometer (top) and (b) the right accelerometer (bottom). The x-axis shows the day number over which the 2D-FT average was calculated, the y-axis is the spectral repetition frequency (Hz) for the mean daily 2D-FT image and the pixel intensity shows the intensity of each frequency of the DVAV signal in arbitrary units, scaled to its maximum every day.

For the data pertaining to the mean daily 2D-FT of the Clifton Observation Hive we see in Fig S20 that the frequency of the spectral repetition of the DVAV signals detected by our software remains stable across the entirety of the recording centred at 19.679 Hz. This is confirmed by simple Linear regression that deduced that the peak frequency of detected DVAV signals cannot be predicted by the day number for the left *(Frequency: R =0.545, p =0.0269)* or the right accelerometer *(Frequency: R =0.035, p = 0.149).* This is in agreement with the data for the French 2015 hive (Fig 8) and French 2017 hive (Fig S21). Due to the reduction in the number of detections by our software during December through until March, the plot appears noisier for these months in S20 Fig. However, the trend is retained even during this period.

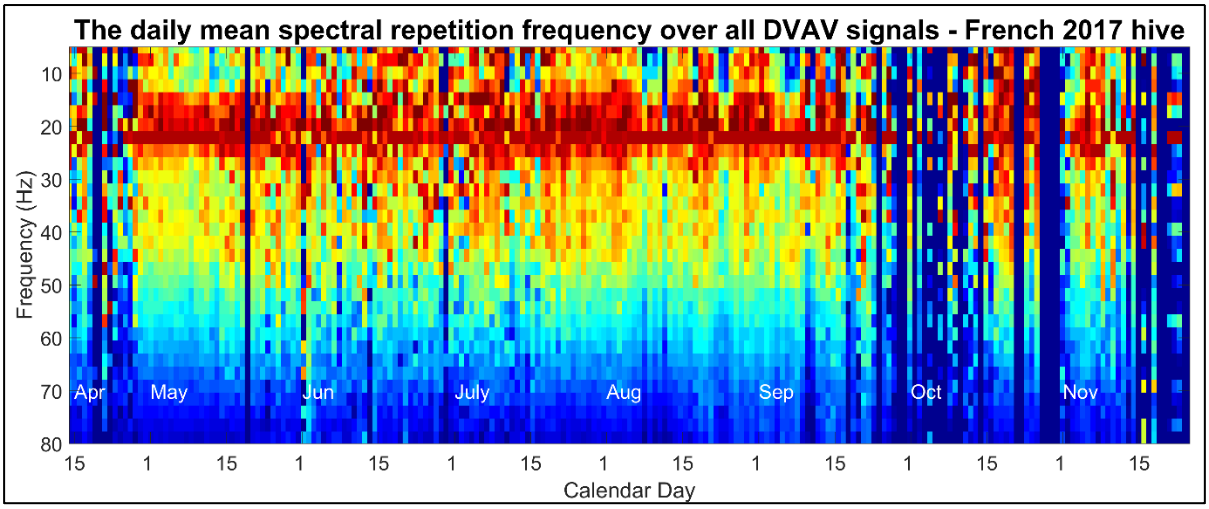

Fig S21. The spectral repetition of the mean 2D-FT (y-axis) computed for all DVAV signals within each day of the recording. The x-axis shows the day over which the 2D-FT average was calculated, the y-axis is the spectral repetition Frequency (Hz) for the mean daily 2D-FT image and the pixel intensity shows the intensity of each frequency of the DVAV signal in arbitrary units, scaled to its maximum every day.

As seen in Fig S21, the frequency of the mean daily spectral repetition of the DVAV signals detected by our software for the 2017 French hive dataset remains stable across the entirety of recording until the colony was found to have collapsed in November. This is confirmed by simple linear regression that deduced that the peak frequency of detected DVAV signals cannot be predicted by the day number for the left *(R =0.523, p =0.0306)*. There was no detection of DVAV signals until the 16th May and therefore the image appears dark blue for this period. After mid-September, the plot becomes more scattered owing to the reduction in the number of detections.

DVAV signal occurrences and weather

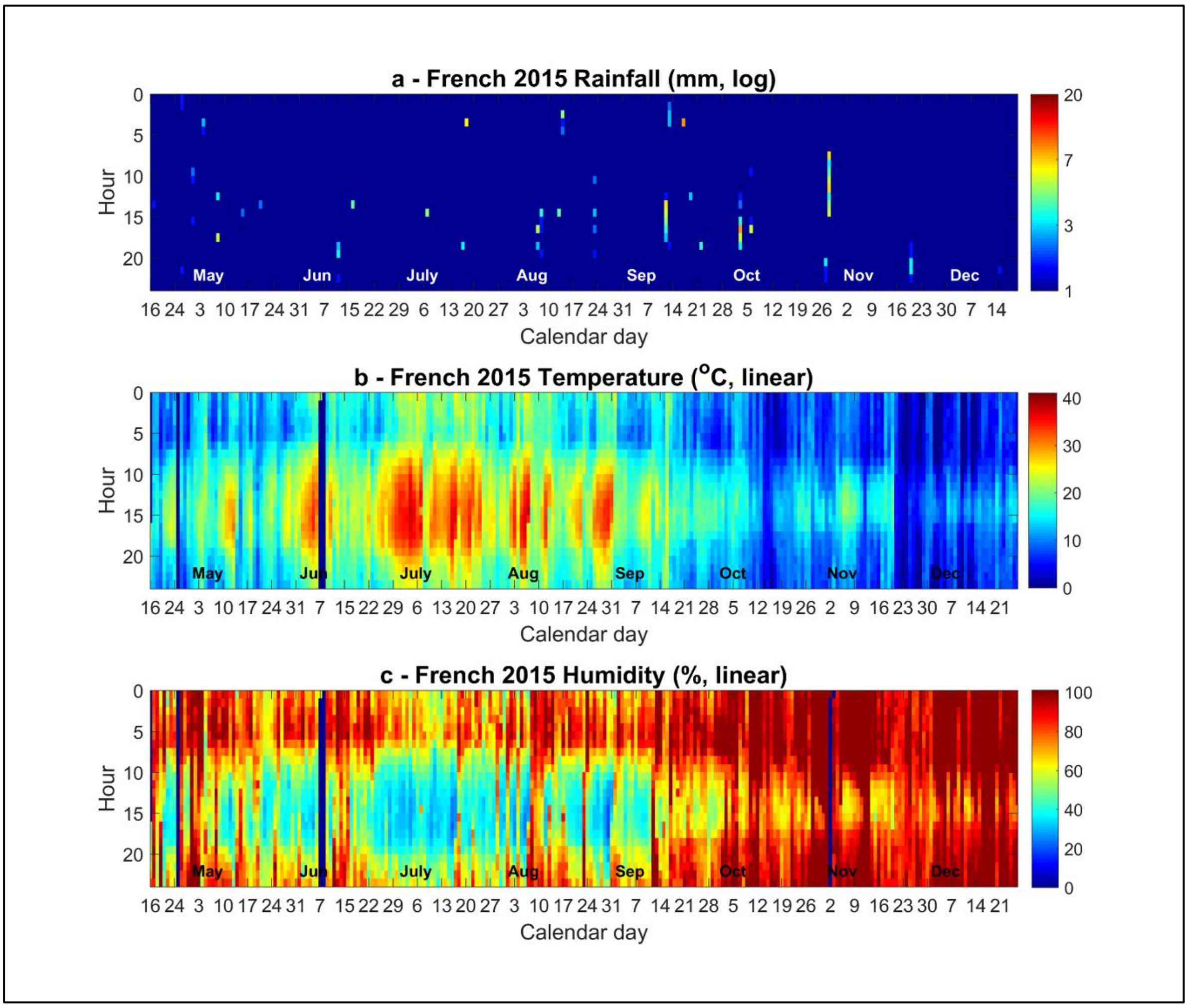

Fig S22. The hourly histograms of rainfall (mm), outside temperature (°C) and humidity (%) plotted for each day that corresponds to and has been formatted to match our histograms of DVAV signal occurrences in Fig 4.

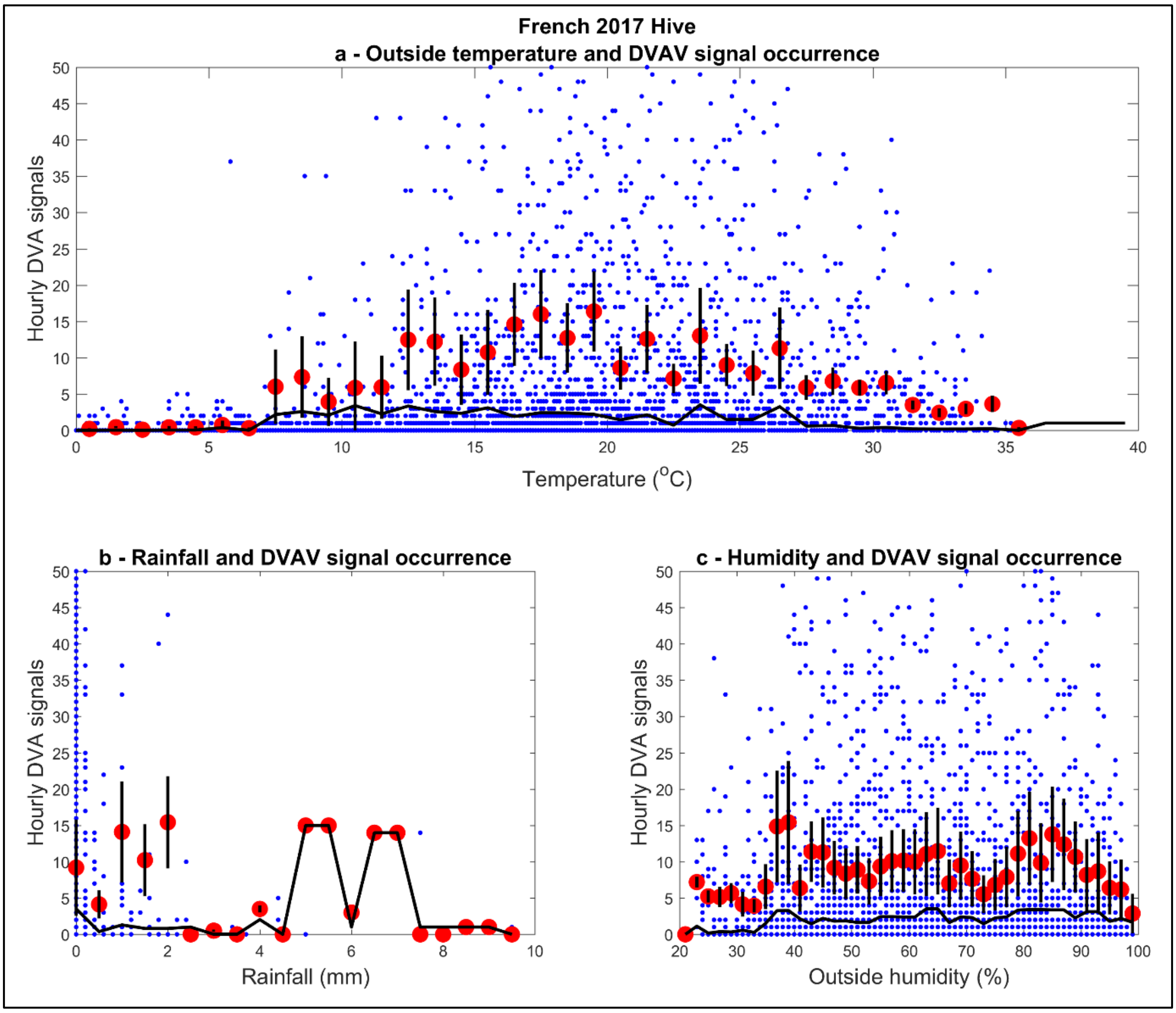

Fig S23. Hourly occurrences of DVAV signals in relation to weather. French dataset (2017 season) with corresponding: (a) average outside temperature, (b) cumulative rainfall, and (c) average outside humidity. Red dots indicate the average number of DVAV signals with black bars displaying ± 1 SE. The black curve on each graph shows the modal hourly DVAV signals.

Plots of the complete hourly data for temperature, humidity and rainfall that co-inside with our 2017 French data recordings (which can be found in Fig S24). It can be seen in Fig S23a that the occurrence of honeybee DVAV signals is at its lowest at the extremes, i.e. at below 7°C and above 35°C, a trend that is mirrored for the 2015 French hive dataset in Fig 9a. There is a steady increase in the occurrence of DVAV signals from 7 to 15°C and then a plateau until 24°C when a steady decline can be seen thereafter. No trend can be seen displayed in the humidity plot in Fig S23c meaning that while temperature and humidity appear to have an inverse correlation in Fig S24b and S24c, they act upon DVAV signals independently. As seen for the 2015 French hive dataset in Fig 9b, there is also no perceivable trend between rainfall and the number of DVAV signals but it can also be seen that the majority of days saw very little precipitation. However, full days of prolonged heavy rain are rare after June 2017, before which, No DVAV signals had been detected.

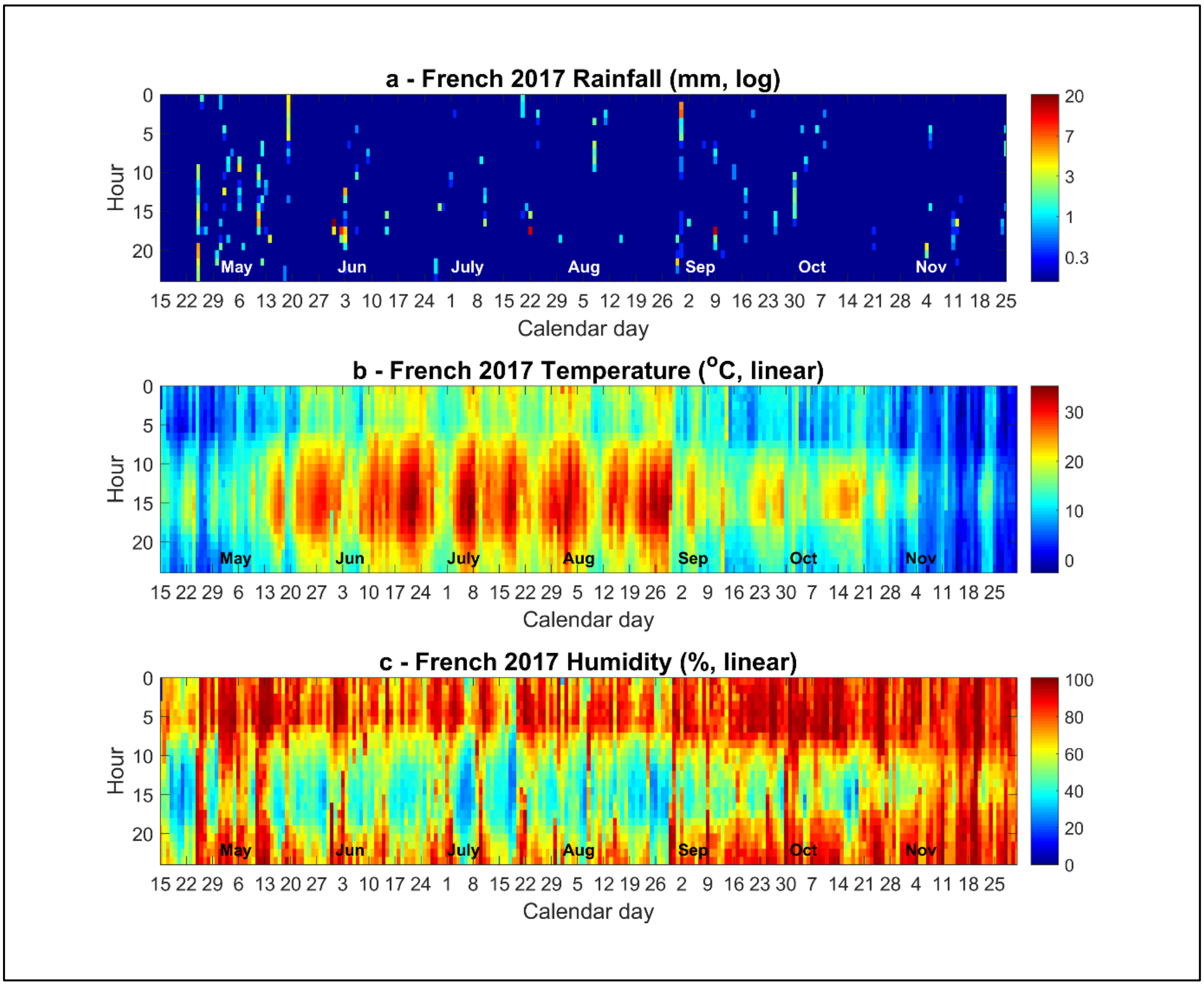

Fig S24. The hourly histograms of rainfall (mm), outside temperature (°C) and humidity (%) plotted for each day that corresponds to corresponds to, and has been formatted to match our histograms of DVAV signal occurrences for the French 2017 hive in supplementary Fig S6a.

Supplementary Methods: Optimisation of the detection software

The first pass.

For the purposes of the vibrational pulse detections required in our study, signal waveforms were categorised as being a member of one of three distinct categories: “DVAV signals”, “Worker Pipes” and a third category called “Noise” that contained spurious high-amplitude sharp-peaked signals. The discrimination software required a two-stage process, because the Discriminant Function Analysis of PCA scores (see Ramsey, et al. [2]) obtained using the information from the 2D-FT of the pulses in the training was found to retain spurious signals within the DVAV signal category. Further inspection by critical listening in combination to visual analysis of the 2D-FT of the detected pulses disclosed that the spurious signal, that we here call “clicks”, exhibited ultra-short peaks similar to those seen in the DVAV signals but without the characteristic temporal periodicity. Therefore, the DVAVs and clicks were further fed into a second discrimination exercise where a slightly different method was required to discriminate between them.

The above categories were subjectively identified after critical listening to the detected pulses from the first-pass without any discrimination exercise. A ‘training database’ for discrimination was constructed using 150 examples of signals, of varying SNR, extracted from this dataset, that were deemed to be highly representative of each category. Each pulse waveform was separately uploaded into the software prior to being manually centred through visual analysis of the position of each waveform within the one-second long window. Each pulse underwent processing whereby the sampling rate was coarsened by a factor 3, the 2D-FT frequencies were cropped vertically and horizontally respectively to 1500 and 70 Hz. The overall amplitude of the resulting cropped 2D-FT was then normalised to one in order to try to detect weak DVAV signals equally as well as strong ones. The data was then reshaped to create a linear array which could later be fed into the PCA/DFA algorithm (for further information see Bencsik, et al. [2]) to identify the features of the signal waveforms that are unique to each category.

The pulse categories were carefully labelled within the training database and their PCA scores calculated. By using a pair of cross correlation products with two discriminant functions identified by the DFA algorithm, two discriminant function coordinates, or ‘DF scores’, could then be calculated for each. The centroids for each group was further calculated. From this, a “threshold value” could be determined for the ratio of distances between the “DVAV signal” centroid to the sum of the distances to the centroids of the other groups. The optimum threshold was that which had the lower percentage error based on how many points from each cluster overlapped with the DVAV cluster. The maximum number of meaningful PCA scores used in the discrimination phase was determined carefully set by avoiding too much numerical noise being fed into the search (Bisele, et al. [3]) To reduce the possibility of over-fitting, an iteration procedure similar to that of Bisele, et al. [3] was also implemented whereby every pulse combination was explored within the training database, removing all pulses except for the collection allowing for clustering with the lowest error. However, pulses removed from the training database that were not included within the computer training stage, were still involved within the clustering process to find their co-ordinates within the DF space (Fig S25). Once the optimum outcome was reached, the coordinates of the centroids of the clusters were stored and software was developed to provide a threshold for determining which pulses would aDVAVnce to the second pass. The best threshold gave an error rate smaller than 1.4%.

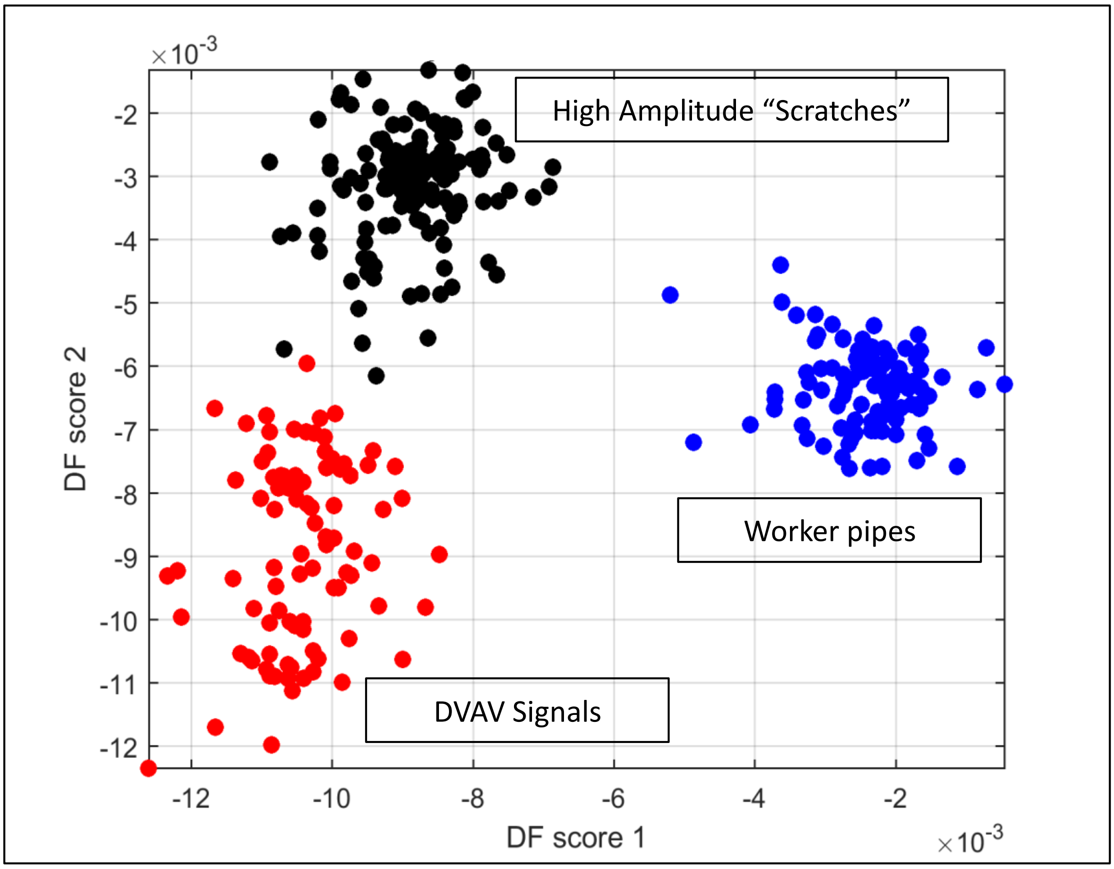

Fig S25. The outcome of the first pass supervised clustering of the groups previously identified as “DVAV signals” (red cloud), “worker piping” (blue clouds) and “noise” (black cloud) for discrimination, shown in two-dimensional DF space. The overlap is negligible and below 1.4 %.

The second pass.

To discriminate true DVAV signals from those comprising of high-amplitude spurious sharp peaks, a protocol similar to the first pass algorithm was implemented with additional information to that used in the first pass. A common source of wrongly detected signals came from high-amplitude “clicking” within the dataset, which comes from sources such as a honeybee “working” on the wax honeycomb using its mandibles as seen in S8 video. When comparing the averaged DVAV knock in Fig S2 to the individual clicks in Fig S26 taken from S8 Video, it is easy to see how the software misidentified these signals in the first pass. The honeycomb does appear to react in a very similar way during honeybee wax chewing or when receiving a DVAV signal, however a rapid oscillation is present at the start of each click waveform that is of low amplitude and high frequency. Highlighted in red in Fig 26: this is probably caused by the honeybee as it grips the honeycomb with its mandibles before pulling at the wax. This additional information was exploited by computing the gradient of the acceleration to help the software to discriminate between DVAV signals and clicks. In addition to the computation of the 2D-FT, as in the second pass, the spectrum of the gradient of the acceleration of the digital signal waveform that had been coarsened by a factor 3, cropped to 1500Hz, normalised to its mean amplitude and finally underwent log transformation was calculated. This information was then reshaped into a linear array and fed into the training database. Optimisation was then achieved by the same methods as for the first pass. The coordinates of the cluster centroids was then computed which

allowed identification of the best threshold for characterisation as a true DVAV signal or a spurious signal (Fig 27).

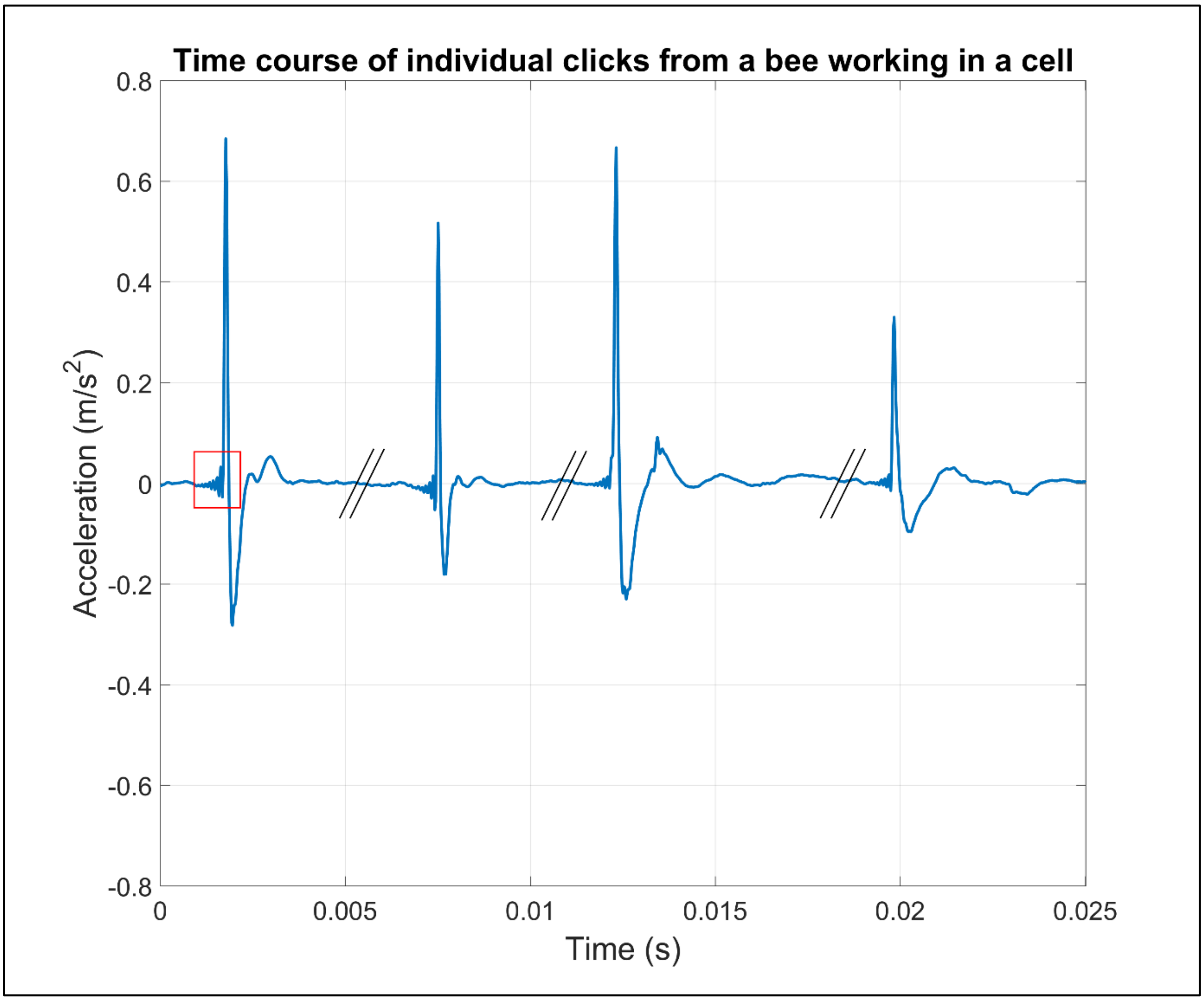

Fig S26. The time course (s) of acceleration (m/s^2) of four individual high-amplitude clicks of similar magnitude that were extracted and concatenated from the calibrated accelerometer data associated with footage of an emerged bee working in a cell (see S8 Video). The symbols // show the points at which the individual clicks were concatenated. The red box highlights a feature unique to this type of signal.

This two-pass discrimination was utilised within the main signal detection software. The fact that only DVAV signals remain after discrimination was thoroughly checked initially by critical listening to hundreds of signals taking place at random times of the year. As critical listening usually fails at identifying a clear audible DVAV signal, this was then further supported by the visual analysis of the 1- and 2- dimensional Fourier transform of the full collection of signals detected by our software. Specific one-hour long sections of data where hot spots of occurrences were detected were also listened to, and indeed revealed highly frequent DVAV signals, as demonstrated in S1 Audio. Thirdly, DVAV signal detections were checked against those seen to take place on the eight hours of video recordings we gathered in synchrony with accelerometer data. The percentage error by which "false positives" were detected by our software even though they are not DVAV signals was around 17%, giving an overall success rate of 83%. A movie of DVAV signals extracted by our software, with the corresponding audio, is also supplied in S4 Video.

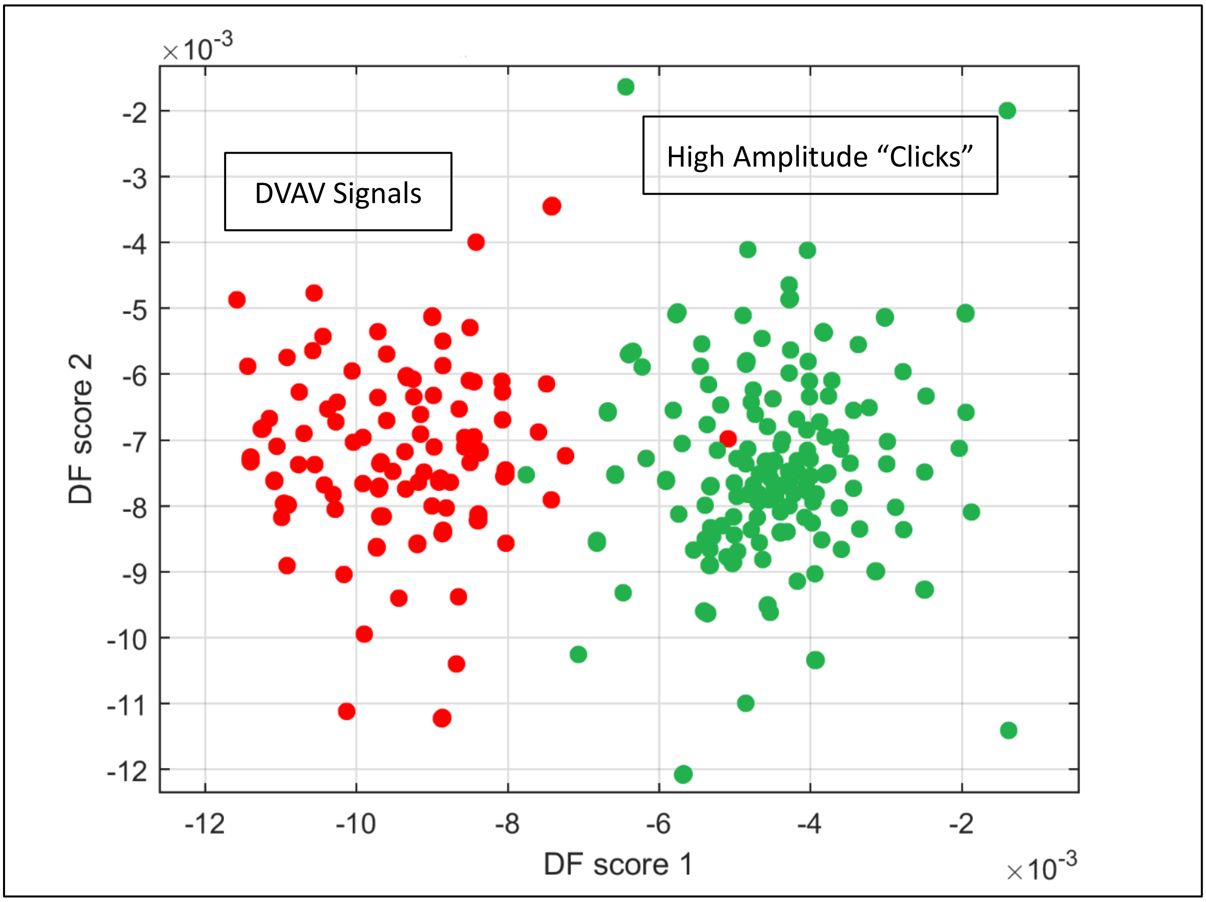

Fig S27. The outcome of the second pass supervised clustering of the groups previously identified as “DVAV signals” (red cloud), and “high amplitude clicks” (green cloud) for discrimination, shown in two-dimensional DF space. The overlap is negligible at below 1 %.

References:

1. Ramsey, M., Bencsik, M. and Newton, M. I. Long-term trends in the honeybee ‘whooping signal’ revealed by automated detection. *PLoS ONE*, 12(2), e0171162, (2017).

2. Bisele, M., Bencsik, M., Lewis, M. G. C. and Barnett, C. T. Optimisation of a machine learning algorithm in human locomotion using principal component and discriminant function analyses. *PLoS ONE.* 12(9), e0183990, (2017).

3. Bencsik M., et al., Honeybee colony vibrational measurements to highlight the brood cycle. *PLoS ONE*. 10(11), e0141926, (2015).
